# Origin and Evolution of Protein Fold Designs Inferred from Phylogenomic Analysis of CATH Domain Structures in Proteomes

**DOI:** 10.1371/journal.pcbi.1003009

**Published:** 2013-03-28

**Authors:** Syed Abbas Bukhari, Gustavo Caetano-Anollés

**Affiliations:** Evolutionary Bioinformatics Laboratory, Department of Crop Sciences, University of Illinois, Urbana, Illinois, United States of America; European Molecular Biology Laboratory, United Kingdom

## Abstract

The spatial arrangements of secondary structures in proteins, irrespective of their connectivity, depict the overall shape and organization of protein domains. These features have been used in the CATH and SCOP classifications to hierarchically partition fold space and define the architectural make up of proteins. Here we use phylogenomic methods and a census of CATH structures in hundreds of genomes to study the origin and diversification of protein architectures (A) and their associated topologies (T) and superfamilies (H). Phylogenies that describe the evolution of domain structures and proteomes were reconstructed from the structural census and used to generate timelines of domain discovery. Phylogenies of CATH domains at T and H levels of structural abstraction and associated chronologies revealed patterns of reductive evolution, the early rise of Archaea, three epochs in the evolution of the protein world, and patterns of structural sharing between superkingdoms. Phylogenies of proteomes confirmed the early appearance of Archaea. While these findings are in agreement with previous phylogenomic studies based on the SCOP classification, phylogenies unveiled sharing patterns between Archaea and Eukarya that are recent and can explain the canonical bacterial rooting typically recovered from sequence analysis. Phylogenies of CATH domains at A level uncovered general patterns of architectural origin and diversification. The tree of A structures showed that ancient structural designs such as the *3-layer (αβα) sandwich (3.40)* or the *orthogonal bundle (1.10)* are comparatively simpler in their makeup and are involved in basic cellular functions. In contrast, modern structural designs such as *prisms*, *propellers, 2-solenoid, super-roll, clam, trefoil and box* are not widely distributed and were probably adopted to perform specialized functions. Our timelines therefore uncover a universal tendency towards protein structural complexity that is remarkable.

## Author Summary

Proteins are vital and central macromolecular players necessary for the functioning of the cell. The redundant and highly conserved structural makeup of proteins reflects their ability to act as genomic repositories of evolutionary history. These structures are fundamental subjects for the study of molecular evolution. Structural biologists have demonstrated the existence of a wide array of compact 3-dimensional fold structures, the protein domains. Their classification resulted in hierarchical taxonomies that describe protein fold space, most notable SCOP, CATH and FSSP. Studies have shown that certain types of protein shapes are more abundant than others and this uneven distribution implicates processes by which new shapes are discovered. Our evolutionary genomic research explores the evolution of protein domains at the deeper levels of classification. However, we have not embarked in a systematic study of the origin and evolution of general structural designs. These designs include topologies such as *sandwiches*, *bundles*, *barrels*, *prisms*, *solenoids*, and *propellers*. The appearance and diversification of general structural designs and their confirmation from published literature defines a unique chronology of structural innovation. The study also uncovers a recent trend of architectural sharing between Archaea and Eukarya and benchmarks the phylogenomic analysis of CATH domains with SCOP domains.

## Introduction

The polypeptide chains of proteins generally fold into highly ordered and well-packed three-dimensional (3D) atomic structures [1]. These protein folds represent spatial arrangements of more or less wound helices (generally α-helices) and extended chain segments (β-strands) that are separated by flexible loops and relatively rigid regions in the form of turns and coils. Helices are stabilized by local main-chain (backbone) hydrogen bonding interactions. In turn, β-strands establish main-chain interactions with other strand elements that are distant. Parallel and antiparallel arrangements of β-strands form β-sheets, which often curve to form open and closed barrel structures. Folds are generally defined by the composition, architecture and topology of their core ‘helix’ and ‘sheet’ secondary structure elements [2]. The satisfaction of the hydrogen bonding potential of main-chains gives rise to regular secondary and super secondary structural elements in globular proteins. Analysis of protein folds indicates that those that occur frequently tend to adopt regular architectures, such as the αβ Rossmann folds, α/β-barrels, β-sandwiches, and bundles [3]. Main-chain hydrogen bonding is also important for the formation of complex turns and coils that link α-helices and β-strands.

**Figure 1:**
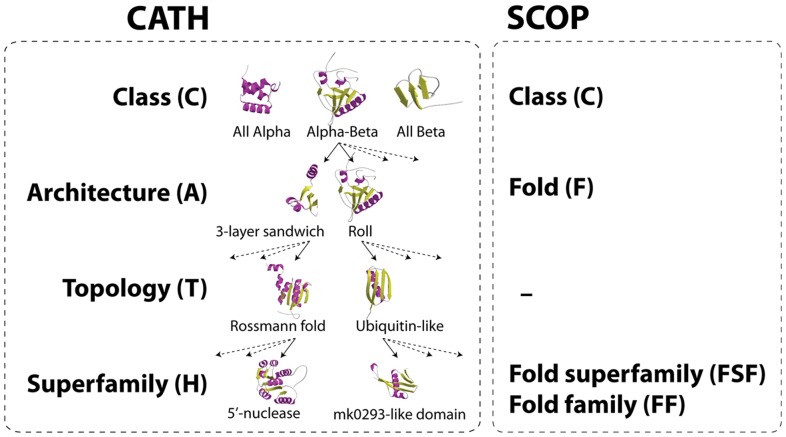
Hierarchy of the CATH structural classification system compared to corresponding SCOP levels. The architecture (A) level is unique to CATH.

Protein domains are compact, recurrent, and independent folding units of protein structure that sometime combine with other domains to form multi-domain proteins. They are considered evolutionary units and are the basis for several protein structure classification schemes. Two of them, CATH and SCOP, are accepted as gold standards and share a number of common features [4]. SCOP [5] is a largely manual collection of protein structural domains that aims to provide a detailed and comprehensive description of the structural and evolutionary relationships of proteins with known structures. In contrast, CATH [6] uses a combination of automated and manual techniques, which include computational algorithms, empirical and statistical evidence, literature review and expert analysis. Both classifications are hierarchical but dissect 3D structure differently, focusing more on either evolutionary or structural considerations [4]. SCOP unifies domain structures that are evolutionarily related at sequence level (>30% pairwise residue identities) and are unambiguously linked to specific molecular functions into fold families (FFs), FFs with common structures and functions with a common evolutionary origin into fold superfamilies (FSFs), FSFs with similarly arranged and topologically connected secondary structures (not always evolutionarily related) into folds (Fs), and finally Fs that share a general type of structure into classes. CATH unifies domain structures hierarchically (bottom-up) into sequence families (SFs; analogous to FFs), homology superfamilies (Hs; analogous to FSFs), topologies (Ts; analogous to Fs), architectures (As), and protein classes [6] (see also Figure 1 for comparisons of SCOP and CATH levels of structural abstractions). Multi-linkage clustering groups domains into SFs based on sequence similarity. SFs with structures that are thought to share common ancestry and can be described as homologous are grouped into Hs. H structures sharing patterns of overall shape and connectivity of secondary structures are grouped into Ts. T structures that share and overall shape of the domain structure according to the orientations of the secondary structures but ignoring their connectivity are unified into As. Finally, A general shapes are grouped into four protein structural classes, mainly-alpha, mainly-beta, alpha-beta and few secondary structures [6].

Protein structures are evolutionarily conserved and capable of preserving an accurate record of genomic history [1], [7]. They represent ‘relics’ of molecular evolution [2] and express the greatest levels of redundancy and reuse that exist in molecular biology [8]. Many studies have been conducted to unfold the evolution and diversification of protein domain structures and proteomes of extant organisms [1], [9]–[11]. Structural phylogenies describing the evolutionary relationship of SCOP F, FSF and FF domains were built by data-mining the census of structures in hundreds of genomes [12]–[15]. Timelines of F, FSF and FF appearance were derived from the phylogenetic trees and revealed the existence of three epochs in protein evolution, ‘architectural diversification’, ‘superkingdom specification’ and ‘organismal diversification’. A universal core of domain structures that is central for cell function was the first to unfold in the timelines during the architectural diversification epoch. During the superkingdom specification epoch, patterns of reductive evolution in the domain repertoire consistently segregated the archaeal lineage from the ancient community of organisms and established a first organismal divide. Finally, the appearance of eukaryotic and archaeal signature domains marked the start of the organismal diversification epoch and the rise of domain structures specific to proteome lineages. Finally, trees of proteomes (i.e. trees of life) placed Archaea at the root and confirmed this organismal supergroup represents the most ancient superkingdom of life [7], [16].

While we have studied how F, FSF and FF domains appeared and distributed in the world of organisms, we have not embarked in a systematic study of the origin and evolution of general structural designs. Here we study how these designs evolve in trees of domain structures, this time focusing on the CATH classification. The appearance and diversification of general protein structural designs at A-level (e.g., *sandwiches*, *bundles*, *barrels*, *solenoids*, *propellers*) and published literature define in this study a unique chronology of structural innovation. Structural phylogenies of domains at T and H levels of structural abstraction uncover global evolutionary patterns of structural distribution in the world of organisms. The study benchmarks previous phylogenetic analysis of SCOP-defined domains and again reveals the early origin of the archaeal superkingdom. Congruent patterns of diversification derived from protein structure provide remarkable support to the ancient history of the cellular world, and trees of life confirm the primordial evolutionary patterns.

## Results/Discussion

### The distributions of CATH domain structures in superkingdoms resemble those of SCOP structures and reveal a consistent evolutionary bias

**Figure 2:**
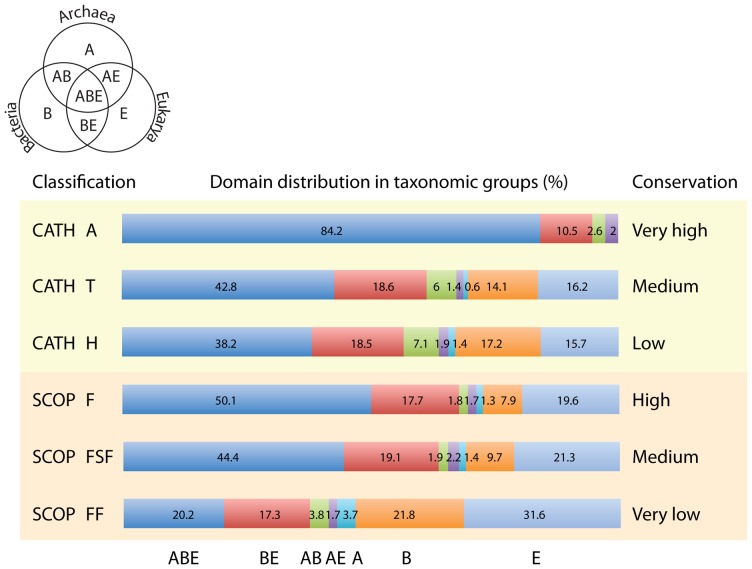
Distribution of CATH domain structures among taxonomical groups of domain distribution in superkingdoms. The percentage of domain structures shared by superkingdoms was considered as coarse estimate of evolutionary conservation of the hierarchical levels of classification. CATH domain censuses were derived from the present study. SCOP values were taken directly from published data [16], [18] and involve 1030 Fs, 1740 FSF and 2,397 FF defined by SCOP v. 1.73.

Domain structures are unevenly distributed in the world of proteins and proteomes [1]. They distribute differently in superkingdoms Archaea (A), Bacteria (B) and Eukarya (E) and can be pooled into seven taxonomical groups depending on whether they are unique to a superkingdom (A, B and E) or are shared by two (AB, AE and BE) or three superkingdoms (ABE). The taxonomical groups can be visualized in a simple Venn diagram (Figure 2). Bias in the relative number of domains structures corresponding to each taxonomical group persists regardless of the classification used (CATH or SCOP) or the level of structural abstraction of the classification scheme (Figure 2). This bias cannot be attributed to non-vertical patterns of inheritance (e.g. the effect of horizontal transfer) since research groups have confirmed independently that convergent evolution is relatively rare (∼2–12%) at these high levels of structural conservation (e.g., [17], [18]).

Distribution biases among taxonomical groups show some striking features. First and as expected, higher taxonomical levels show higher levels of structural sharing between superkingdoms (especially ABE) than lower taxonomical levels, confirming the contention that they are evolutionarily more conserved. Second, the highest level of structural abstraction (CATH A) does not contain a single superkingdom-specific taxonomic group, suggesting that these groups represent sets of structures that are late evolutionary additions to the protein repertoire. Finally, ABE and BE domain structures are consistently the dominant taxonomic groups at all hierarchical levels, from FF to A. This final observation suggests they represent the most ancestral and common taxonomical groups. The most parsimonious corollary of these evolutionary patterns of domain distribution is that the ancient BE taxonomical group must arise by loss of archaeal-specific domain structures, suggesting Archaea is the most ancient superkingdom. As we will now show, this suggestion can be confirmed by phylogenomic reconstruction.

### Structural chronologies of CATH domain structures uncover patterns of proteome diversification

**Figure 3:**
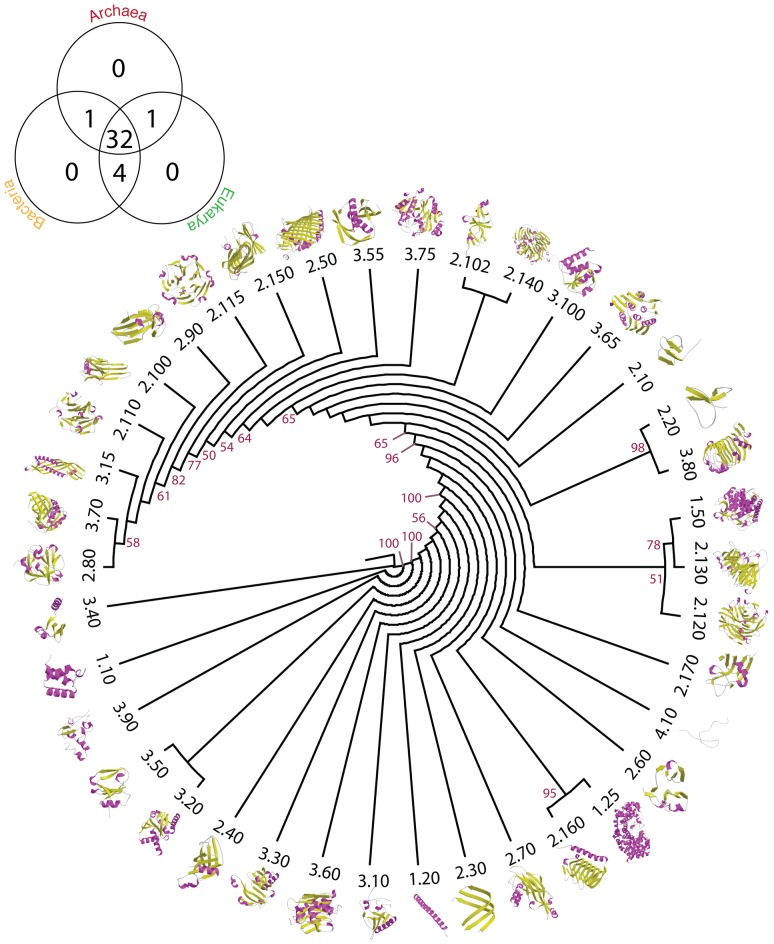
Phylogenomic tree of CATH A domain structures. Optimal (P<0.01) most parsimonious A (26,323 steps; CI = 0.3738, RI = 0.7655; g1 = −0.427) tree was reconstructed from a protein domain census in 492 completely sequenced genomes. The phylogeny was plotted into circular tree diagram and cartoon representations of the core structures labeled with each CATH id were mapped onto the leaves of the tree. The Venn diagram shows the diversity of A in the three superkingdoms, Archaea, Bacteria and Eukarya.

**Figure 4:**
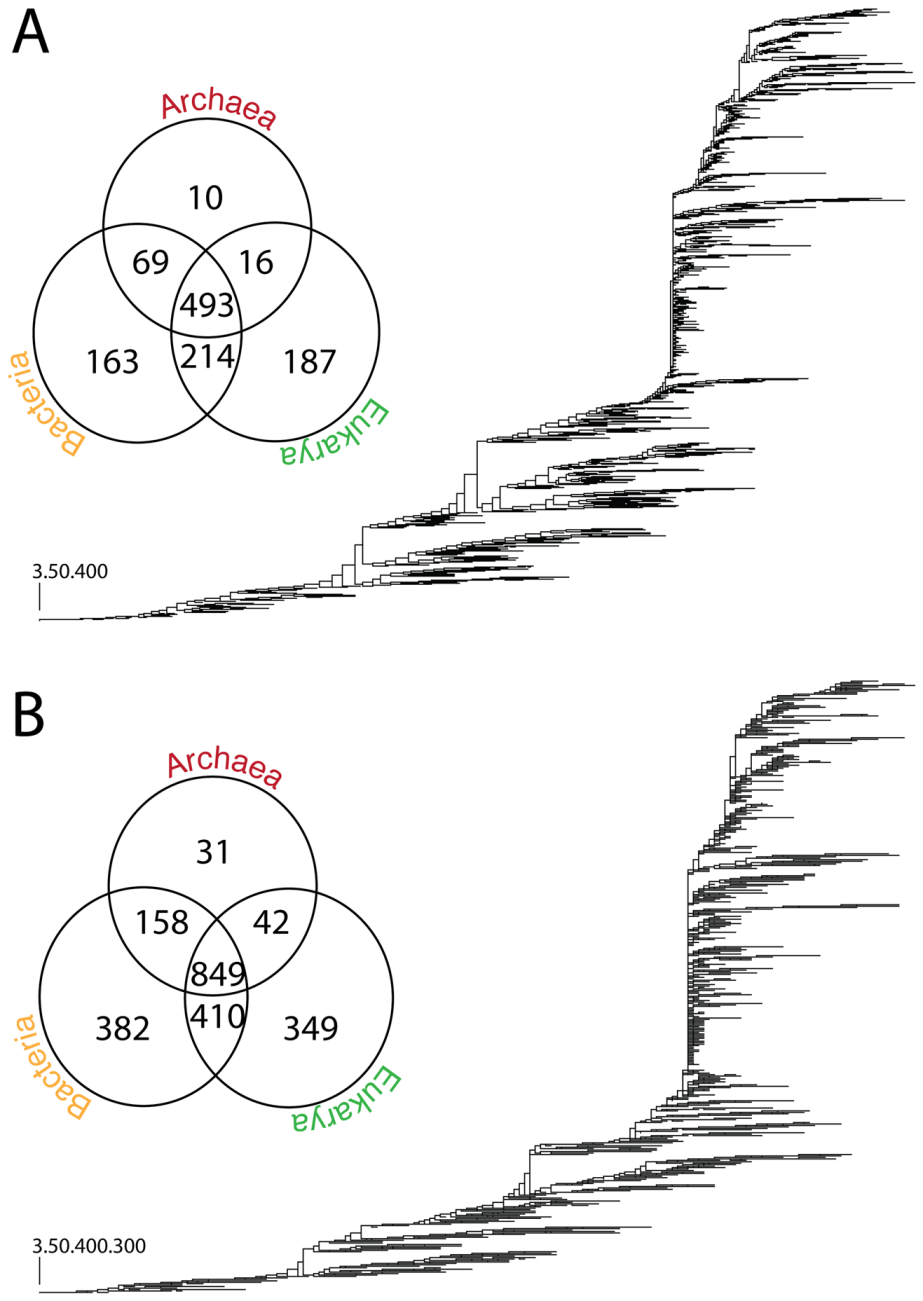
Phylogenomic trees of CATH T (A) and H (B) domain structures. Optimal (P<0.01) most-parsimonious T (392,769 steps; CI = 0.0251, RI = 0.7488; g1 = −0.169) and H (658,425 steps; CI = 0.0149, RI = 0.7444; g1 = −0.144) trees were reconstructed from a protein domain census in 492 completely sequenced genomes. The phylogenies reconstructed from a genomic census of 1,152 Ts and 2,221 Hs in 492 proteomes, where all 492 characters were parsimoniously informative. Terminal leaves are not labeled because they would not be legible. The Venn diagram shows the diversity of Ts and Hs in the three superkingdoms, Archaea, Bacteria and Eukarya.

**Figure 5:**
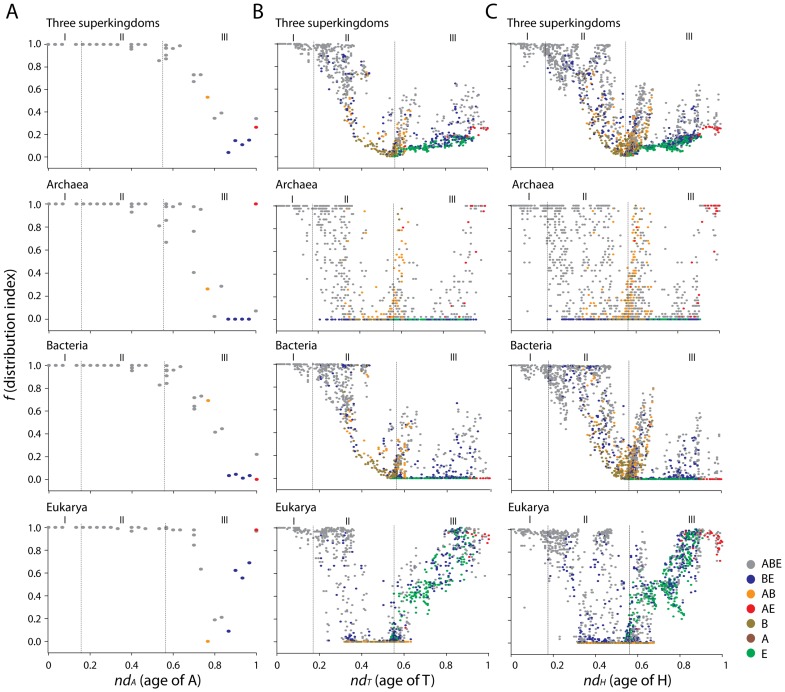
Architectural chronologies of CATH A, T and H domain structures. Three phases or epochs (I, II and III) in the timeline delimit the appearance, crystallization and diversification of As (**A**), Ts (**B**) and Hs (**C**) in all three superkingdoms (top panels) and in Archaea, Bacteria, and Eukarya (bottom panels). Individual plots show the relationship of *f* (distribution Index) and age of domain structures defined at A (*nd_A_*), T (*nd_T_*) and H (*nd_H_*) levels of structural abstraction.

We generated phylogenomic trees describing the phylogenetic relationship of 38 A, 1,152 T and 2,221 H domain structures (Figures 3 and 4). Tree distribution profiles and metrics of skewness suggest significant cladistic support (P<0.01). The trees were well resolved. However, internal branches for trees of Hs and Ts were poorly supported by bootstrap analysis, an expected outcome with trees of this size. Chronologies of evolutionary appearance [7] of CATH domain structures were derived directly from the phylogenomic reconstructions. The relative age of domains (*nd*) was measured on the trees as a relative distance in nodes from the hypothetical ancestor of domains at the base of the trees, and used to build the timelines. Since our method produces rooted trees that are highly unbalanced and reject the Yule and random speciation models [19] and since molecular speciation in our trees has clock-like behavior and does not depend on changes in domain abundance [20], *nd* was considered a good and most-parsimonious proxy for time. To study how domain structures distribute in proteomes, we calculated a *distribution index* (*f*), the number of species that use each structure given on a relative 0–1 scale. The *f* index was plotted along the timelines of domain structures, i.e. against *nd* (Figure 5). Three As (*nd_A_* = 0–0.068), fifteen Ts (*nd_T_* = 0–0.061) and fifteen Hs (*nd_H_* = 0–0.049) were present in all proteomes examined (*f* = 1) and were the most ancient in the timeline. A list of the fifteen Hs is given in Table S1. The *f* of As decreased with increasing age. The *f* of Ts and Hs decreased with their increasing age until *f* approached zero at *nd_T_* = 0.55 and at *nd_H_* = 0.55, respectively. We term these ages “crystallization points” of the T and H structural chronologies, borrowing the idea of a phase transition from physics. At these time points, a steady decrease in *f* results in a large number of structures being specific to a small number of organisms. After crystallization, an opposite trend takes place, in which Ts and Hs increase their representation in genomes. In contrast, the architectural chronology that describes the appearance of As remained unaffected by the crystallization event since the losing trend of As started at *nd_A_* = 0.56–0.60 but rarely reached zero (Figure 5).

**Figure 6:**
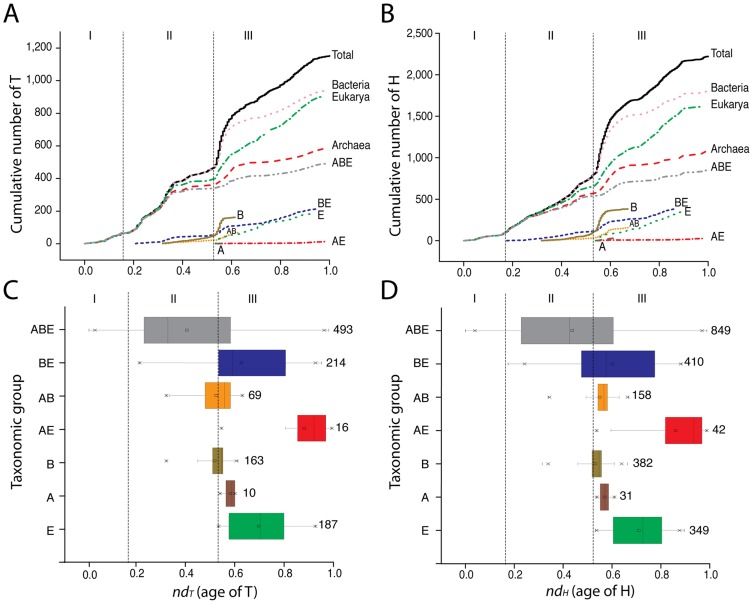
Cumulative frequency plots of CATH H and T domain structures. Cumulative frequency distribution plots plotted against the respective for T (**A**) and H (**B**) domain structures. Bottom plots show boxplots describing *nd* ranges for the seven taxonomic groups of T (**C**) and H (**D**) structures that are unique to individual superkingdom (A, B, E) or shared by two (AB, BE, AE) or all (ABE) superkingdoms. Numbers of T and H structures belonging to each taxonomic group are also indicated.

To uncover hidden patterns of organism diversification in our dataset, we divided structures according to their distribution in superkingdoms and constructed three separate structural chronologies for the genomes of each superkingdom at A, T and H levels of structural abstraction (Figure 5). Taxonomical groups of domain structures were identified in the time plots with different colors. We previously observed that a superkingdom must ‘lose’ a significant number of SCOP structures before the evolutionary appearance of the first superkingdom-specific ‘signature’ structure [7]. In our study, this loser trend of domain structures was also observed for the CATH annotated genomes in each superkingdom. This observation strengthens our claim of reductive evolution in protein domains of the lineages that emerge from the cellular urancestor (the last universal common ancestor; LUCA) that we find is functionally complex [11]. The loser trend of SCOP and CATH structures reveals the primordial birth of Archaea followed by the birth of Bacteria and Eukarya. For example, the complete loss of Hs first starts in Archaea (*nd_H_* = 0.176) with the membrane-bound lytic murein transglycosylase D (chain A) H domain (*3.10.350.10*). Its appearance is congruent with the loss of the first SCOP FSF in Archaea (*nd_FSF_* = 0.174), the *LysM domain* (d.7.1), observed in previous studies [7]. Both domain definitions are very much similar in how they describe functions in the cell. Analysis of domain distribution in Archaea shows that the vast majority of ancient Ts and Hs that were lost in proteomes were present in all superkingdoms (ABE; colored grey). These were followed by AB (orange), A (wine) and few AE (red) structures, most of which started to appear after the crystallization point and during the superkingdom specification and organismal diversification epochs [7]. Clear decreases in structural representation (*f*-value) also occurred in Bacteria and Eukarya, but involved fewer and younger structures. Analysis of domain distribution in Bacteria shows that AB and B structures (dark yellow) started to increase representation after the crystallization point, leading towards their diversification and specification. Similarly, the eukaryotic chronology showed that comparatively younger architectures [e.g. BE (blue) and E (green)] increased their popularity among the eukaryal lineages. The appearance and distribution of the seven taxonomical groups of T and H structures was unfolded in the timelines using boxplots describing the range of *nd_T_* and *nd_H_* values and measures of central tendency for each group (Figure 6). Only domains shared by the three superkingdoms (ABE) span the entire chronology, from the origin of proteins (*nd* = 0) to the present (*nd* = 1). These structures represent instantiations of the domain content of the urancestor but their late appearance may also indicate events of horizontal transfer between lineages. Boxplots for BE, AE and AB explain relationships among superkingdoms over time. The BE boxplot is the most ancient of the three, suggesting Archaea diversified early by reductive evolution. The A, B and E boxplots reflect the history of ‘signature’ structures that are unique to individual superkingdoms. These signatures appear first in Bacteria and then concurrently in Archaea and Eukarya, an observation that is congruent with timelines derived from SCOP domains [7]. Despite its early specification, Archaea tends to acquire Archaea-specific structures very late in evolution and their number is limited when compared to Bacteria and Eukarya. This may stem from very strong adaptive pressures that were historically imposed by lifestyle. Archaea are very simple organisms that usually live in harsh and extreme environments [21]. We believe their extremophilic lifestyles impose constraints on their molecular make up that: (i) limit the possibility of acquiring new structures, and (ii) induce a constant selective pressure to maintain a minimal structural set necessary for survival. We therefore propose that Archaea maintained a minimal set of structures while losing structures by strong reductive evolution. We note that signature As exhibit very low *f* values, suggesting these molecular designs were acquired as adaptations to new environments and lifestyles. The appearance of structures shared by only two superkingdoms was also revealing. For example, the AE boxplot's upper whisker approached *nd_H_* = 1, implying a recent relationship between Archaea and Eukarya. Comparatively, the *nd* values for SCOP FSFs for the AE taxonomical group was *nd_FSF_* = 0.85, supporting the late appearance of the interaction [7]. Note that a sister relationship between Archaea and Eukarya is usually used to claim the canonical bacterial rooting of the tree of life [22], but that in our studies this relationship is only supported by domain structures that are quite derived. It is also noteworthy that the early loser trend in the BE taxonomic group, made explicit by smooth decreases in f-values in the timeline, occurs in the absence of signature domain structures specific to superkingdoms (Figure 5). This weakens other evolutionary scenarios of superkingdom origin, including chimerism mediated by massive horizontal gene transfer (endosymbiosis or fusion) processes, and the possibility that phylogenetic signal of these events (e.g. those between Bacteria and Eukarya) would make Archaea appear artificially ancient in phylogenomic reconstructions (see below).

### Trees of proteomes derived from the CATH genomic census confirm the early emergence of Archaea

**Figure 7:**
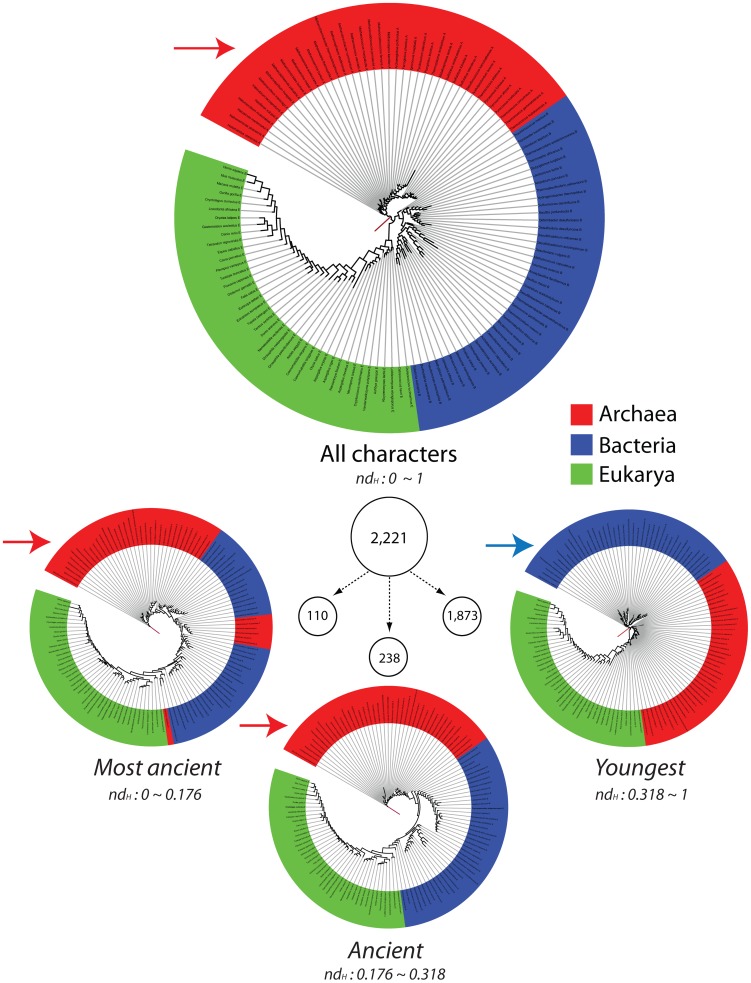
A phylogenomic tree of proteomes generated from the equally sampled dataset of FL proteomes. The circular cladogram of the most parsimonious rooted tree describes the evolution of 123 equally sampled proteomes and was generated from genomic abundances of 2221 Hs. Terminal nodes of Archaea (A: 41 proteomes), Bacteria (B: 41), and Eukarya (E: 41) were labeled in red, blue, and green, respectively. Also the total character set was divided into three independent character sets e.g. Most Ancient (*nd_H_* 0∼0.176), Ancient (*nd_H_* 0.176∼0.318) and Younger (*nd_H_* 0.318∼1) characters set. These character sets resulted in three trees of proteomes that reflected the behavior of the tree over different character sets. Root branches are indicated with arrows.

We previously reconstructed trees of proteomes from a genomic census of SCOP domains and made inferences about the rooting of the tree of life [7], [11], [16]. We found trees of proteomes reconstructed from ancient domain structures were rooted paraphyletically in Archaea while trees reconstructed using derived structures exhibited the canonical rooting with Bacteria emerging at their base. We also revealed how parasitic and symbiotic lifestyles can complicate phylogenetic interpretation [7], [16]. The proteomes of organisms that are parasitic or that establish symbiotic relationships with other organisms have frequently experienced reductive evolution, discarding enzymatic and cellular machineries in exchange for resources from their hosts. Since their inclusion can lead to incorrect phylogenetic trees, we excluded proteomes from all but 295 free-living (FL) organisms and reconstructed rooted trees that most parsimoniously describe their evolution. The FL set included 41 archaeal, 189 bacterial, and 65 eukaryotic organisms. The tree of FL proteomes reconstructed from a census of H domain structures supported the trichotomy of the superkingdoms (Figure 7). The number of bacterial proteomes was however overrepresented in the FL-tree and could cause long-branch attraction during phylogenetic reconstruction possibly leading to incorrect deep phylogenetic relationships. Since taxon sampling can also affect phylogenomic inference [23], we randomly sampled equal numbers of proteomes per superkingdom (a maximum of 41) and generated replicated trees of proteomes. Reconstruction of equally sampled FL proteomes improved tree resolution and bootstrap support values of deep branches. More importantly, the trees consistently showed a paraphyletic rooting in Archaea and the derived placement of monophyletic Bacteria and Eukarya (Figure 7). We also reconstructed trees of FL proteomes from three subsets of phylogenetic characters: ancient H structures common to all superkingdoms corresponding to the architectural diversification epoch (*nd_H_*<0.176), H structures of intermediate ancestry corresponding to the superkingdom specification epoch (0.176<*nd_H_*<0.55) and H structures that are derived and reflect the organismal diversification epoch (0.55<*nd_H_*). The proteome tree reconstructed from the most ancient H structures was rooted paraphyletically in Archaea, reflecting their early segregation through the minimalist strategy. Reconstructions from H structures of intermediate ancestry produced trees with three clades corresponding to the three superkingdoms that were rooted in Archaea. Finally, reconstructions from H structures that were derived yielded the canonical tree of life rooted in Bacteria. It is noteworthy that the rooting of these trees reflects the early appearance of Bacteria-specific domain structures (Figure 7, see trees reconstructed using most ancient, ancient and younger characters sets). We note the split of Archaea in three groups in the tree reconstructed from ancient H structures. We believe this anomaly stems from using subsets of characters in phylogenomic reconstructions and from the existence of a ‘modern effect’ [11] imposed by relatively recent changes in abundance of domain structures belonging to the ABE taxonomic group. Both factors impoverished phylogenetic signal and obscured deep phylogenetic relationships. The modern effect is an embodiment of recent evolutionary processes affecting ancient repertoires, the effects of which must be identified and removed when reconstructing the set of domain structures present in the urancestor [11].

### Modern Archaeo-Eukaryotic architectural sharing questions the canonical tree of life

**Figure 8:**
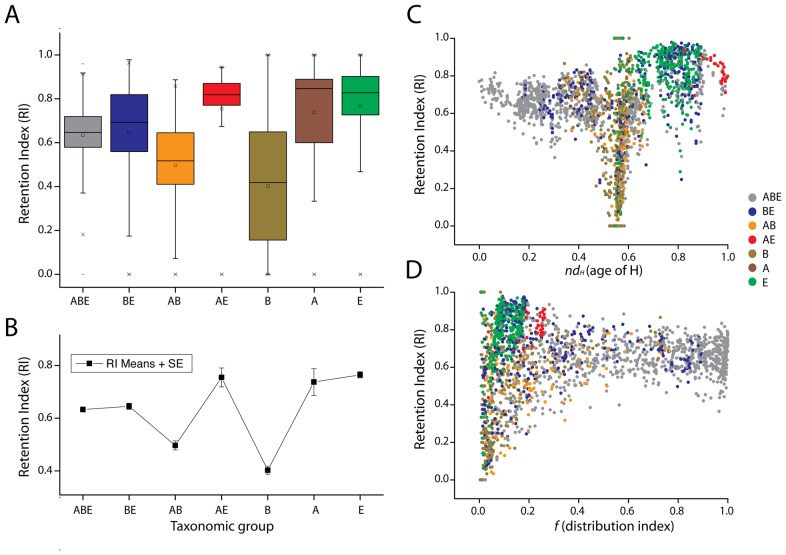
The extent of synapomorphy exhibited by phylogenomic characters (H) in the trees of proteomes. (**A**) Boxplots for retention index (RI) values of characters specific to seven taxonomical groups. (**B**) Mean RI for each taxonomical group was plotted with its standard error. (**C**) RI is plotted against the age (*nd_H_*) of each character, colored according to its specific taxonomical group. (**D**) RI is plotted against the f distribution index of each, same coloring scheme were used as of (**C**).

The structural chronology, especially at H level, unveils a relatively recent (perhaps ongoing) sharing of protein architectures between archaeal and eukaryal genomes. The timeline reveals that while AE domain structures appeared for the first time when Archaea and Eukarya acquired their superkingdom-specific signature structures, the vast majority of them appeared quite late in evolution (e.g., Figure 6D). This was unanticipated. This finding inspired us to resolve the phylogenetic contribution of each structural character set in the tree of proteomes. Interestingly, characters that are shared by archaeal and eukaryal genomes exhibited high retention index (RI) values (Figure 8), indicating that the sharing pattern did not result from annotation artifacts. The RI measures the amount of synapomorphy (features that are shared and derived) expected from a data set that is retained as synapomorphy on a cladogram. Boxplots of structural character sets shared by the seven taxonomical groups were also plotted (Figure 8). Since low RI values indicate high levels of homoplasy (i.e. non-vertical phylogenetic signal), the low values of bacterial signature structures confirm the high incidence of horizontal gene transfer that exists in the bacterial superkingdom. In turn, the relatively high RI levels of the common ABE group is surprising. Most members of the group include very ancient structures (Figure 8), many of which were part of the urancestor. High RI levels in this taxonomical group challenge the common assumption that horizontal transfer was rampant during early life [22].

These RI boxplots are powerful enough to explain the relationships of superkingdoms in our tree of proteomes. The AE boxplot is the only one exhibiting very high RI values. In turn, bacteria-specific characters had the most dispersed RI boxplot. Hence, archaeal and eukaryotic lineages share good signal characters that are very recent and are widely present; their high *f* values indicate for example their presence in most of archaeal and eukaryotic proteomes (Figure 5C). More than 30 years ago, Woese and Fox [24] defined the existence of three ‘aboriginal’ lines of descent – superkingdoms Archaea, Bacteria and Eukarya. The microbial Archaea and Bacteria lines were conceptualized as ‘urkingdoms’ of deep origin that were qualitatively different from the eukaryotic kingdoms. This prompted reconstructions of a tripartite tree of life and later proposals of the early rise of Bacteria with rooting determined using paralogous gene couples (e.g., EF-Tu/EFG). This classical (canonical) tree topology induces sister lineages corresponding to Archaea and Eukarya and an exclusive common ancestor of both. Many archaeal components involved in informational systems (e.g. translation, replication and transcription) and transmission of genetic information show a higher sequence similarity with their eukaryotic homologue than their bacterial homologue [25], [26]. For instance, more than 30 ribosomal proteins are shared between the Archaea and Eukarya that are not present in Bacteria [27]. Moreover, Archaea and Eukarya also share a similar base excision repair system that is different than the system in bacteria [28]. If the phylogenetic signal in the sequence of these RNA and protein molecules adequately depicts history, these findings would explain the evolutionary link between Archaea and Eukarya and the topology of the canonical tree of life that emerges in some phylogenetic studies from their close relationship. However, many genes do not share the archaeal and eukaryal link and the canonical root must be considered contentious. Remarkably, the tree of proteomes reconstructed using the modern structural character set in our experiments (Figure 7, epoch III or younger character sets) is the only tree with the canonical topology that places the root branch in Bacteria. This topology mostly results from protein domain structures of very recent origin that are shared between Archaea and Eukarya. We contend that these very recent domains retain good phylogenetic signal, especially in their sequences, and will be less affected by processes of mutation saturation. Consequently, the close evolutionary relationship of Archaea and Eukarya in trees of life derived from analyses of these sequences [22], [24] can be considered an artifact of the focus on sequence.

Current trees of life built for example from sequence concatenation, such as those in refs. [29], [30], include genes encoding for multidomain proteins (e.g. aminoacyl-tRNA synthetases). Some of these domains are of recent origin and may fall within the derived domain set we have analyzed. We claim that strong phylogenetic signal in the sequence of these domains likely drives the reconstructed topologies. Instead, weak phylogenetic signal embedded in the sequences of older and universal domains is swamped by the recent archaeo-eukaryotic signal that is in part responsible for the canonical tree. Our focus on CATH domain structure (not gene sequence) can dissect the differential contribution of old and recent protein domains that belong to the proteome-encoding gene repertoire. A similar focus on deep phylogenetic signal in RNA structure has also shown the basal placement of Archaea in phylogenetic reconstructions from tRNA, RNase P RNA and 5S rRNA [31]–[35], including analysis of paralogy in tRNA [35]. For example, a timeline of accretion of helical RNA substructures of RNase P complexes showed the most ancient substructures were universal and harbored the core catalytic activities of the endonuclease [34]. However, the first substructures that were lost were specific to Archaea and this episode occurred before molecules were accessorized with superkingdom-specific substructures. The early origin of Archaea was also shown in trees that describe the structural evolution of RNase P RNA, which placed archaeal molecules at its base. These results obtained by studying the evolution of RNA structure clearly parallel the evolutionary patterns of CATH domain accumulation of this study. Clearly, deep phylogenetic signal in protein and RNA structure is free from the limitations of gene sequence and associated non-vertical patterns arising from horizontal gene transfer but more importantly from domain rearrangement and can therefore reveal historical patterns without bias [36]. Here we show the importance of considering the age heterogeneity of a biological repertoire, in this case the proteome, when making phylogenetic statements.

### Chronologies of CATH architectures reveal evolutionary patterns of structural diversification

The architectural chronology of As is evolutionarily more conserved than chronologies of Hs and Ts (Figure 5A). The timeline shows that As are widely shared and are refractory to loss in genomic lineages. In fact, very few As are lost in superkingdoms (4 in Archaea, and one each in Bacteria and Eukarya) and are thus very old and popular in the world of organisms. The *3-layer (αβα) sandwich (3.40)* is the most abundant and ancient of all proteins. The *orthogonal bundle (1.10)* and the *α/β-complex (3.90)* are equally abundant and are the second and third most ancient architectures. Remarkably, the phylogenomic tree of As shows that comparatively simpler shape structural designs are more favored than complex designs and in general are more ancient, appearing at the base of the tree. Architectural complexity was here evaluated on empirical grounds by focusing on the topology and regularity of spatial arrangements of secondary structures in a structural design. For example, the most ancient *3.40* and *1.10* architectures involve simple arrangements of secondary structure that can be very diverse in different structural variants while more recent shape designs are spatially more convoluted and regular (Figures 3). As time progresses the complexity in architectural make up of structural designs also increases (Figure 3). The few As that are lost in superkingdoms are quite complex and as expected their appearance is quite derived. The first loss occurred in Eukarya (*nd_A_* = 0.76) with the very complex *Clam* architecture, and then in Archaea and Bacteria. We note that Archaea loses four As quite late and in a row, showing that the pervasive reductive trends of Archaea described above extend almost to the present. This also reflects the conservative nature of extremophilic Archaea, which are not in need of modern structural designs. Bacteria loses the most recent A structural design, *Box* (*2.80*), at *nd_A_* = 1, which is shared by both archaeal and eukaryal genomes. *Box* is involved in nucleotide excision repair, a molecular function that has a unique place in cellular defense because of its wide substrate range and its ability to virtually remove all base lesions from a genome. Ögrünç et al. [28] reported a similar base excision repair system used in Archaea and Eukarya and argued that a different set of proteins are employed by the bacterial nucleotide repair system. Interestingly, the *f* index for *Box* in Archaea (*f* = 1) and Eukarya (*f* = 0.96) again indicates a recent sharing of structural designs between archaeal and eukaryal organisms. Architectures constitute the second highest level of structural abstraction in CATH, and because of their high conservation it is difficult to clearly delimit the three epochs of the protein world. In contrast, our results indicate CATH H and SCOP FSF are the most suitable levels to uncover the evolution of domain structures in genomes. These levels of abstraction are structurally and evolutionarily conserved. They preserve deep phylogenetic signatures and are variable enough to dissect evolutionary history of proteomes and molecular functions.

### CATH architectures become more complex in evolution

**Figure 9:**
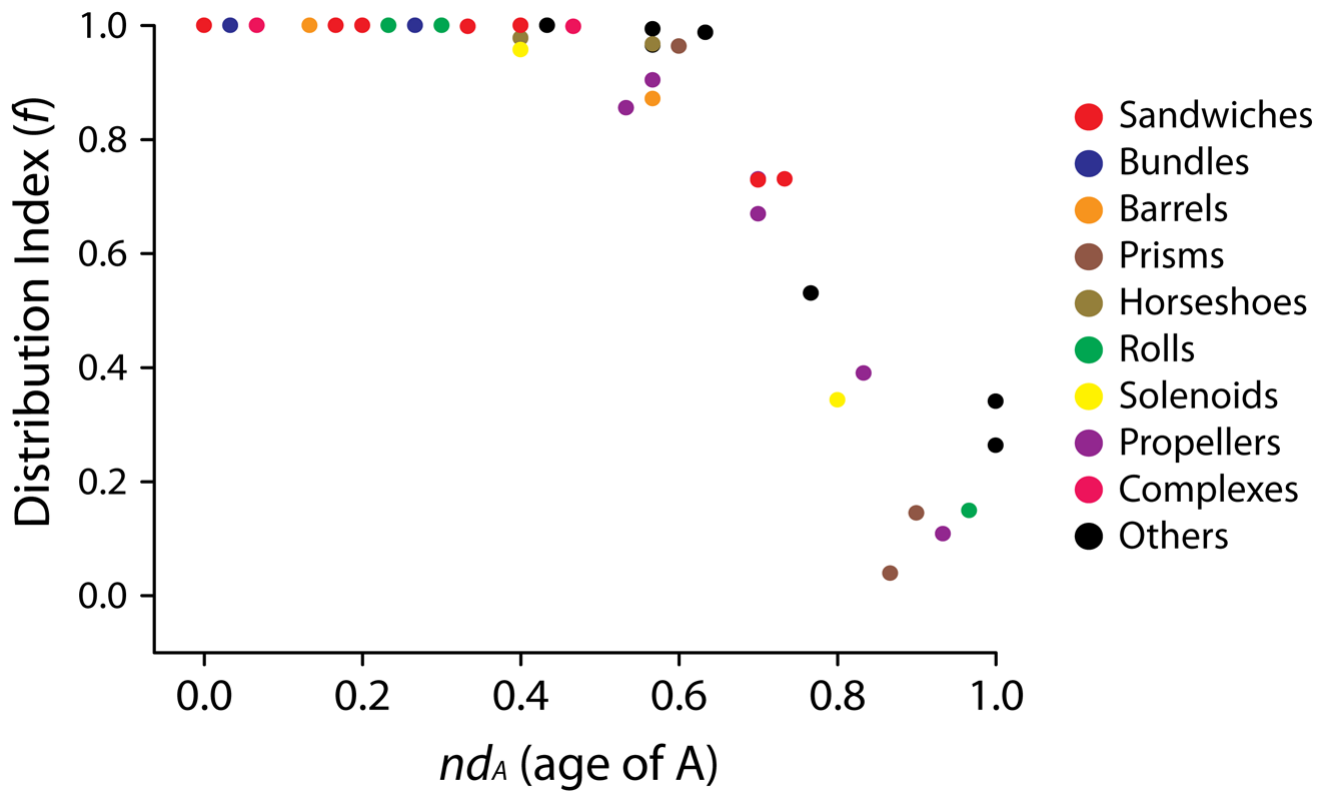
Architectural chronologies of CATH A domain structures colored according to structural design. As shown in Table 1 we grouped the 38 As into 10 larger sets of general structural designs. As were plotted against their age (*nd_A_*) and *f* distribution indices, whereas each A was colored according to their general structural design group.

**Figure 10:**
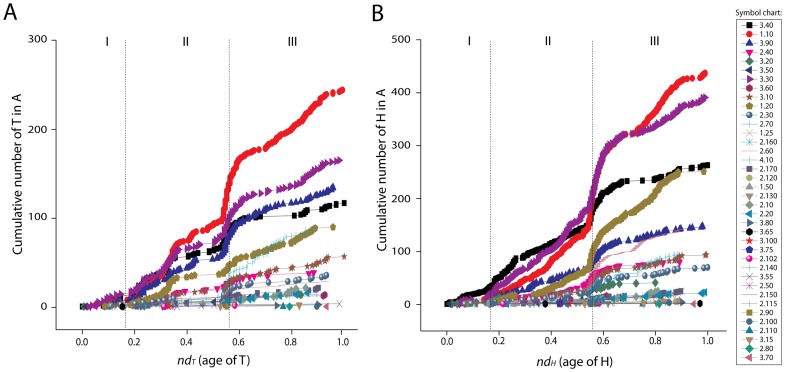
Cumulative frequency distributions of Ts and Hs belonging to a particular A along timeline of domain structures. Plots A and B describe the evolutionary appearance of T and H domain structures, respectively. These two plots uncover patterns of diversification of structural designs in architectures over time. For example, the evolutionary accumulation of Ts and Hs belonging to the oldest architecture, the 3-layer (αβα) sandwich (3.40), occurs early but at different rates than Ts and Hs belonging to the orthogonal bundle (1.10) and 2-layer sandwich (3.30). The same pattern can be seen in (B), where the accumulation of the 4-layer sandwich (1.20) surpasses that of the α-β complex (3.90), even if 3.90 is more older than 1.20.

To obtain a detailed view of architectural discovery and usage over time, we grouped As into 10 major structural designs: *sandwiches*, *bundles*, *barrels*, *prisms*, *horseshoes*, *rolls*, *solenoids*, *propellers*, *complexes* and *other* (a category with structural designs that could not be clearly grouped into the main categories) (Table 1 and Figure 9). We found that most *sandwiches*, *bundles*, *barrels*, *complexes* and *rolls* have high *f* values (*f*∼1) and rather simple structural designs (Figure 9). In turn, structural designs such as *propellers*, *horseshoes*, *solenoids (2 Solenoid, 2.150), prisms, trefoil* and *box*, have low *f* values (*f* = 0.85–0.10) and are very complex. Under the assumption that widespread and abundant designs are old, complex folds appear to have evolved later than simpler folds. We also mapped the appearance of T and H structures harboring individual A designs, plotting *nd_H_* and *nd_T_* values for Hs and Ts belonging to each of the 38 known As (Figure 10).

**Table 1:** Grouping of CATH A level structures into 10 general categories.

*Index*	*CATH A ID*	*CATH A Description*	*nd_A_*	*f_A_*	*General Design*	*Group*
1	3.40	3-Layer (αβα) Sandwich	0	1	Sandwich	ABE
2	1.10	Orthogonal Bundle	0.03	1	Bundle	ABE
3	3.90	α-β Complex	0.06	1	Complex	ABE
4	2.40	β Barrel	0.13	1	Barrel	ABE
5	3.20	α-β Barrel	0.13	1	Barrel	ABE
6	3.50	3-Layer (ββα) Sandwich	0.13	1	Sandwich	ABE
7	3.30	2-Layer Sandwich	0.16	1	Sandwich	ABE
8	3.60	Up-down Bundle	0.2	1	Bundle	ABE
9	3.10	Roll	0.23	1	Roll	ABE
10	1.20	4-Layer Sandwich	0.26	1	Sandwich	ABE
11	2.30	Roll	0.3	1	Roll	ABE
12	2.70	3 Solenoid	0.33	0.99	Solenoid	ABE
13	1.25	Distorted Sandwich	0.4	0.97	Sandwich	ABE
14	2.160	Sandwich	0.4	0.95	Sandwich	ABE
15	2.60	α Horseshoe	0.4	1	Horseshoe	ABE
16	4.10	Irregular	0.43	1	Others	ABE
17	2.170	β Complex	0.46	0.99	Complex	ABE
18	2.120	6 Propeller	0.53	0.85	Propeller	ABE
19	1.50	α/β Barrel	0.56	0.87	Barrel	ABE
20	2.130	Ribbon	0.56	0.90	Others	ABE
21	2.10	Single Sheet	0.56	0.99	Others	ABE
22	2.20	7 Propeller	0.56	0.96	Propeller	ABE
23	3.80	α-β Horseshoe	0.56	0.96	Horseshoe	ABE
24	3.65	α-β Prism	0.6	0.96	Prism	ABE
25	3.100	Ribosomal Protein L15	0.63	0.98	Others	ABE
26	3.75	3-layer Sandwich	0.7	0.73	Sandwich	ABE
27	2.102	8 Propeller	0.7	0.72	Propeller	ABE
28	2.140	5-stranded Propeller	0.7	0.66	Propeller	ABE
29	3.55	3-Layer (βαβ) Sandwich	0.73	0.73	Sandwich	ABE
30	2.50	Clam	0.76	0.53	Others	AB
31	2.150	2 Solenoid	0.8	0.34	Solenoid	ABE
32	2.115	5 Propeller	0.83	0.38	Propeller	ABE
33	2.90	Orthogonal Prism	0.86	0.03	Prism	BE
34	2.100	Aligned Prism	0.9	0.14	Prism	BE
35	2.110	4 Propeller	0.93	0.10	Propeller	BE
36	3.15	Super Roll	0.96	0.14	Roll	BE
37	2.80	Trefoil	1	0.34	Others	ABE
38	3.70	Box	1	0.26	Others	AE

The table describes the age (*nd_A_*), *f* distribution index, general structural designs, and taxonomical groups for the 38 CATH A in our dataset with two-letter CATH code and keyword description.

The structural makeup of the most ancient *3-layer (αβα) sandwich (3.40)* architecture (Figure 3) represents the central theme of the most ancient SCOP FFs [37]. These structures consist of repeating α-β-α supersecondary units, such that the outer layer of the structure is composed of helices packing against a central core of parallel β-sheets. Many enzymes, including most of those involved in glycolysis, are α/β layered proteins and are cytosolic [38]. These α/β structures harbor repeats of the α-β-α arrangement (e.g., the α-β-α-β-α sequence). The β-strands are parallel and hydrogen bonded to each other, while the α-helices are all parallel to each other but are antiparallel to the strands. Thus the helices pack against the sheet forming a sandwich-like structure. We note that the β-α-β-α-β (αβα) subunit, often present in nucleotide-binding proteins, represents the *Rossmann* structural motif found in proteins that bind nucleotides, especially the cofactor NAD(H) [39].

The *orthogonal bundle (1.10)* and *α-β-complex (3.90)* appear immediately after the *3-layer (αβα) sandwich (3.40)* design. The *orthogonal bundle* consists of a 3–4 α-helix bundle and is found in a number of different proteins, most of which associate with membranes. Due to physical constraints imposed by the lipid bilayer of membranes the list of possible membrane protein structures is limited to either bundles [40], [41] or barrels [42], [43]. In many cases the α-helices are part of a single polypeptide chain and are connected to each other by three loops. In the 4-helix bundle proteins the interfaces between the helices consist mostly of hydrophobic residues while polar side chains on the exposed surfaces interact with the aqueous environment. A number of cytokines consist of 4-helix bundles, such as *interleukin-2*, *interleukin-4, human growth hormones*, and the *granulocyte-macrophage colony-stimulating factor (GM-CSF)* [38] and DNA binding proteins (e.g., transcription factors, repressors proteins) [44]. CATH has grouped the complex shaped structures into the ‘complex’ bin, until alternative assignment methods are developed. The *α/β-complex* architecture groups together all those designs that include significant α and β secondary structural elements in a mixed fashion. Examples of *α/β-complex* proteins include bacterial and mammalian *pancreatic ribonucleases* [45], *Zn metallo-proteases* and *DNA topoisomerases* [46]. Two kinds of *barrel* structures are the most ancient and abundant in the protein world, the *α/β-barrel (3.20)* and the *β-barrel (2.40)* [6], and both appeared at about the same time (*nd_A_* = 0.13). The *α/β-barrel* is composed of eight α-helices and parallel β-strands that alternate along the peptide backbone. The *α/β-TIM barrel* is the most prominent example of *α/β-barrel* and is widely present in enzymes of central metabolism [47]. A *β-barrel* is a large β-sheet that twists and coils to form a closed structure in which the first strand is hydrogen bonded to the last. *β-*strands in *β-barrels* are typically arranged in an antiparallel fashion. Barrel structures are commonly found in porins and other proteins that span cell membranes and in proteins that bind hydrophobic ligands in the barrel center, such as *lipocalins* [48]. The *roll* is a complex nonlocal structure in which 3–4 pairs of antiparallel β-sheets, only one of which is adjacent in sequence, are ‘wrapped’ in 3D space to form a barrel shape [49]. Rolls appear for the first time at *nd_A_* = 0.3.

A number of distinct and more complex architectures appear later on in the chronology, including *solenoids, horseshoes, prisms, propellers and trefoils*. *Solenoid* proteins, with their arrays of repeating motifs, tend to have elongated structures that contrast with the majority of globular proteins whose polypeptide chains follow more complex trajectories [50]. These are constructed from tandem structural repeats arranged in superhelical fashion, a feature that is important for many cellular processes [51]. Solenoid proteins constructed from HEAT repeats [52] and *armadillo* repeats [53], [54] constitute the principal transport receptors. A key structural property that differentiates solenoid proteins from other structured proteins is the lack of contacts between distal regions of protein sequence (sequence-distal contacts). For this reason, solenoid proteins are often more flexible than other structured proteins and this flexibility is an important feature of their specific functions [50]. The solenoid structure appears for the first time at *nd_A_* = 0.46. The *α-horseshoe* protein appears at *nd_A_* = 0.4, is a super helical structure made up of a number of three α-helical orthogonal bundle repeats. The α-β horseshoe appeared at *nd_A_* = 0.56, consists of several α/β-repeating units [55]. The structure of the *ribonuclease inhibitor*, a cytosolic protein that binds strongly to any *ribonuclease* that may leak into the cytosol, takes the concept of the repeating α/β unit to the extreme [55]. The structure is made of a 17-stranded parallel β-sheet curved into an open horseshoe shape, with 16 α-helices packed against the outer surface. *Prisms* are similar to *solenoids* in geometry but completely different in connectivity. A more self-contained β-sheet forms each face of a triangular prism. They appear late at *nd_A_* = 0.86. The *trefoils* consist of an unusual β-sheet formed by six β hairpins arranged with three fold symmetry into ‘Y’ like structures [56] and are also quite derived (*nd_A_* = 1).

### Models of evolution of CATH and SCOP domain structures are congruent

The most ancient and popular architecture, the *3-layer (αβα) sandwich* (*3.40*), harbors the most ancient and abundant topology, the *Rossmann fold* (*3.40.50*) and the most ancient and abundant superfamily, the *P-loop containing nucleotide triphosphate hydrolases* (*3.40.50.300*). Despite differences of topology and ranking within databases [57], this H structure of CATH is analogous to the “*P-loop containing nucleotide triphosphate hydrolase*” FSF (c.37.1) of SCOP [4], since both have Rossmann fold topology and also agree on their keyword definitions. A careful analysis of CATH and SCOP structures phylogenies show that the ancient domains structures at T (3.40.50) and H (3.40.50.300) levels are in global agreement with timelines of F (c.37) and FSF (c.37.1) structures [7]. Despite differences in domain definitions of tertiary structure in CATH and SCOP, the remarkable conservation of evolutionary signal indicates both classification systems effectively preserve evolutionary information in protein structure and uncover global patterns of origin and diversification that are for the most part congruent.

We note that levels of structural abstraction above H and FSF unify domains that may not be necessarily homologous. In other words, T and A in CATH and F in SCOP may show episodes of structural convergence. This could complicate evolutionary interpretations. The fact that the same evolutionary patterns observed using H domain structures in this study (and FSF structures in previous studies; reviewed in [1]) could be recovered at higher levels of the structural hierarchy is encouraging and suggests that the influence of convergent processes at these higher levels is limited and that the classifications do in general a good job in capturing true evolutionary information.

### Major conclusions

In this study we follow the history of protein fold structures and proteomes in the tripartite world of organisms. Instead of generating trees of life from protein sequence with standard methods, we use a genomic structural census and robust cladistics methods to build trees of domain structures and proteomes. Structural phylogenies describing the evolution of CATH domains at A, T and H levels of structural abstraction revealed patterns of reductive evolution and the three epochs in the evolution of the protein world that were previously proposed [7]. Structural diversification patterns match those observed in the analysis of SCOP domain structures [7], [16], [58]. Reconstruction of phylogenomic trees of proteomes describing the evolution of lineages confirms Archaea is the most ancient superkingdom.

Provided assumptions of our phylogenomic method are considered valid, six major findings summarize novel results and take advantage of the ability of CATH to better describe topological features of protein structure:

1. Structural designs that are architecturally simpler are ancient and highly abundant in the extant world of proteins and organisms. We find the *3-layer (αβα) sandwich* cytosolic architecture and the *orthogonal bundle* that often associates with membranes are the most ancient and are preferentially involved in metabolic activities. The origin of proteins thus lies at the interface of primordial membranes and cytoplasm. Bundles and barrels that populate membranes soon follow. Metabolic and membrane proteins thus appear crucial for the early biochemistry of primordial cells [37].
2. Structural designs that are architecturally complex, such as *prisms*, *propellers, 2-solenoid, super-roll, clam, trefoil and box* are derived and less favored in the world of organisms. These designs are generally specific to groups of organisms and have been probably adopted for specialized functions.
3. Although CATH and SCOP differ significantly in their protein domain definitions and in the hierarchical partitioning of fold space, we find that both protein structural classifications classify a protein on very similar theoretical grounds by taking into account their structural, functional and evolutionary roles. Remarkably, CATH and SCOP structures harbor similar phylogenetic signatures and reveal patterns of origin and diversification that are congruent.
4. Structural chronologies provide evidence that Archaea established the first organismal divide by losing a substantial number of domain structures early in evolution. We speculate this reductive evolutionary process reflects the environmental pressure of an ancient extremophilic lifestyle that forced maintenance of a minimal domain repertoire. Our analysis provides additional support to the archaeal rooting of the tree of life obtained from evolutionary analysis of SCOP domains (e.g. [7], [16]), RNA structure [31]–[34], tRNA paralogs [35], and a phylogenomic analysis of abundance of Gene Ontology terms in functiomes (Kim, Nasir and Caetano-Anollés, ms. submitted).
5. Structural chronologies uncover a recent trend of sharing of domain structures between Archaea and Eukarya that continues to the present and involves complex architectures such as the *Box* (2.80) design that is involved in nucleic acid repair.
6. Finally, we also speculate that this modern archaeo-eukaryotic architectural sharing pattern is the most probable reason for the bacterial rooting of the canonical tree of life reconstructed from changes in sequence. In contrast to structure, sequence evolution is more dynamic and prone to phylogenetic signal loss [36]. It is therefore likely that most useful phylogenetic signal in these sequence studies is drawn from structures that have been developed quite recently in evolution.

We note that these conclusions entrust CATH with the ability to properly apportion domain structures in fold space and are only valid if assumptions of character argumentation are valid.

Our trees of domain structures define timelines that trace back the history of innovation, diversification and distribution of protein structural designs. Our finding that protein architectures tend to become more complex in evolution is very significant. In a previous study, analysis of β-barrel structures revealed that the curl and stagger and complexity of the connectivity of supersecondary structures increases in evolution [12]. The very early appearance of multilayered sandwich structures is also compatible with the finding that the most ancestral folds share a common architecture of interleaved β-sheets and α-helices [12]. An even more recent study shows that 36 out of the 54 most ancient FFs harbor α/β/α-layered sandwich structures [37]. The very early appearance of the P-loop hydrolase motif in the first FF, the ABC transporters, was associated with a built-in lateral bundle, which resembles the trans-membrane domains of transporter proteins. This suggests that first proteins contained sandwich and bundle structures and were associated with the membranes of primordial cells. Remarkably, P-loop hydrolase folds and bundles make up important membrane complexes, such as ion channels and transporters. Their very early origin highlights a crucial links between the origin of proteins and the origin of cells.

## Materials and Methods

Phylogenomic trees describing the evolution of domain structures and proteomes were reconstructed using a census of domain abundance in proteomes using PAUP* version 4.0b10 [59]. Figure S1 presents a flowchart of the methodology. CATH annotations for the proteomes of 492 fully sequenced genomes (42 Archaea, 360 Bacteria and 90 Eukarya) were retrieved from *Gene3D* [60]. We used CATH version 3.3 and its corresponding Gene3D assignments. Table S2 lists the organisms studied and Table S3 lists the subset that is free-living and was used in phylogenomic analyses. *Gene3D* is a repository of manually curated HMM predictions with a false positive prediction rate of only 0.2–0.6%. As with *SUPERFAMILY* [9], [61], a repository of SCOP domain predictions, proteomes deposited in *Gene3D* were searched against HMM libraries using the iterative Sequence Alignment and Modeling System (SAM) method. Data matrices of genomic abundance (*G*) of domains at A, T and H levels were assembled for phylogenetic analysis. Empirically, *G* values represent numbers of multiple occurrences of an A, T and H domain in a genome, ranging from 0 to thousands and resembling morphometric data with large variances. Because existing phylogenetic programs can process only tens of phylogenetic character states depending on user's CPU performance, the space of *G* values in the matrix was reduced using a standard gap-coding technique with the following formula:
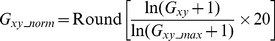
in which 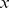 denote either an A, T or H domain structure, 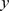 a genome, and 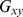 the abundance of 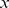 in 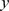. 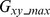 indicate maximum 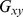 values for all 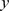 genomes. The round function normalizes *G* values on a 0–20 scale (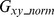). These values define character states, which are encoded as linearly ordered multistate phylogenetic characters using an alphanumeric format of numbers 0–9 and letters A–K that is compatible with PAUP*. A ‘by hand’ generic example of data normalization and encoding is shown in Protocol S1. The actual raw matrix describing the A-level domain census is shown in Dataset S1 as an example. Transposition of the data matrix (switching characters and taxa) allowed reconstruction of trees of either proteomes or domain structures. Trees of A, T and H domains were built by polarizing states from ‘K’ to ‘0’ using the ANCSTATES command in PAUP*, with ‘K’ being ancestral. Trees of proteomes were built by polarizing character states from ‘0’ to ‘K’, with ‘0’ being ancestral. The trees were rooted without invoking outgroup taxa using the Lundberg method, which positions the most ancient proteomes and domain structures at the base of their corresponding trees. Assumptions of character argumentation have been discussed in previous publications [1], [7], [12], [15]. Our model of structural evolution (‘K’ to ‘0’ polarization) considers that the abundance of individual domain structures increases progressively in nature, even when expanding domain levels suffer loss in individual lineages or are selectively constrained during evolution (we consider that character state transformation is reversible). Consequently, ancient structures are more abundant and widely present than younger ones. In contrast, our model of proteome evolution (‘0’ to ‘K’ polarization) assumes proteomes have built their structural repertoires progressively, increasing both the diversity and abundance of their structural make up.

While character argumentation considers that domain structures that appear early in evolution are prominent in genomes and that their numbers increase in steps corresponding to the addition or removal of a homologous gene in a family, the model is agnostic about how changes occur. For example, duplications of domains with simple structural motifs that occur in multiples may involve the entire array, and if these tandem duplicates confer selective advantage, they can be retained in the lineage and can distribute throughout proteome lineages. This is the case for example with proteins that contain tandem repeats of several domains from a same family that are common in Eukarya [62]. While this mechanism of domain gain in not accounted by the model, evolutionary statements relate to domain taxa and their definitions, which generally consider domains as structural and evolutionary modular units.

Phylogenomic trees were reconstructed using the maximum parsimony (MP) optimality criterion in PAUP* with 1,000 replicates of random taxon addition, tree bisection reconnection (TBR) branch swapping, and maxtrees unrestricted. Phylogenetic confidence was evaluated by the nonparametric bootstrap method with 1,000 replicates (resampling size matches the number of the genomes sampled; TBR; maxtrees, unrestricted). The degree of phylogenetic signal for taxa was measured using the skewness (*g_1_*) test with a tree length distribution obtained from 1,000 random trees.

Since trees of domain structures are rooted and are highly unbalanced, we unfolded the relative age of protein domains directly for each phylogeny as a distance in nodes (node distance, *nd*) from the hypothetical ancestral architecture at the base of the trees in a relative 0–1 scale. *nd* was calculated by counting the number of internal nodes along a lineage from the root to a terminal node (a leaf) of the tree on a relative 0–1 scale with the following formula:
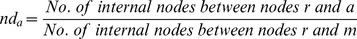
where *a* represents a target leaf node (either an A, T or H domain), *r* is a hypothetical root node, and *m* is a leaf node that has the largest possible number of internal nodes from node *r*. Consequently, the *nd* value of the most ancestral taxon is 0, whereas that of the most recent one is 1. Node distance can be a good measure of age given a rooted tree because the emergence of protein domains (i.e., taxa) is displayed by their ability to diverge (cladogenesis or molecular speciation) rather than by the amount of character state change that exists in branches of the tree (branch lengths).

In this study we have not compared phylogenies recovered using different versions of CATH. However, our experience with SCOP definitions over the years has shown that tree topologies do not change significantly and that evolutionary inferences stand despite biases in the databases [8] and addition of new domain structures to the known repertoire of proteins [1]. We note that the atomic structures of most protein folds have been acquired (∼1,200 out of 1,500 expected)[63]. Consequently, new domain structures are by definition either rare in genomes or intrinsically difficult to recover. Since important evolutionary patterns obtained using CATH definitions match those derived from SCOP, we do not expect CATH updates will change the central conclusions of our study.

## Supporting Information

Dataset S1 A-level genomic abundance matrix.
(TXT)

Figure S1 A methodological flowchart illustrating the reconstruction of phylogenies of proteomes and protein architectures using protein domain census data.
(PDF)

Protocol S1 By hand example of protein domain data normalization and encoding. The objective is to build a phylogenomic matrix for the reconstruction of a tree of domain structures.
(PDF)

Table S1 List of 15 most ancient and popular Hs.
(PDF)

Table S2 List of 492 organisms with their genome id and genome names.
(PDF)

Table S3 List of 295 FL proteomes used to reconstruct proteome trees.
(PDF)
