# Supplementary material for: Origin and Evolution of Protein Fold Designs Inferred from Phylogenomic Analysis of CATH Domain Structures in Proteomes
Source: PLoS Comput Biol. 2013 Mar 28;9(3):e1003009. doi: 10.1371/journal.pcbi.1003009 (PMC3610613; doi:10.1371/journal.pcbi.1003009)
Supplement: Figure S1 — A methodological flowchart illustrating the reconstruction of phylogenies of proteomes and protein architectures using protein domain census data. (PDF) [file pcbi.1003009.s002.pdf]

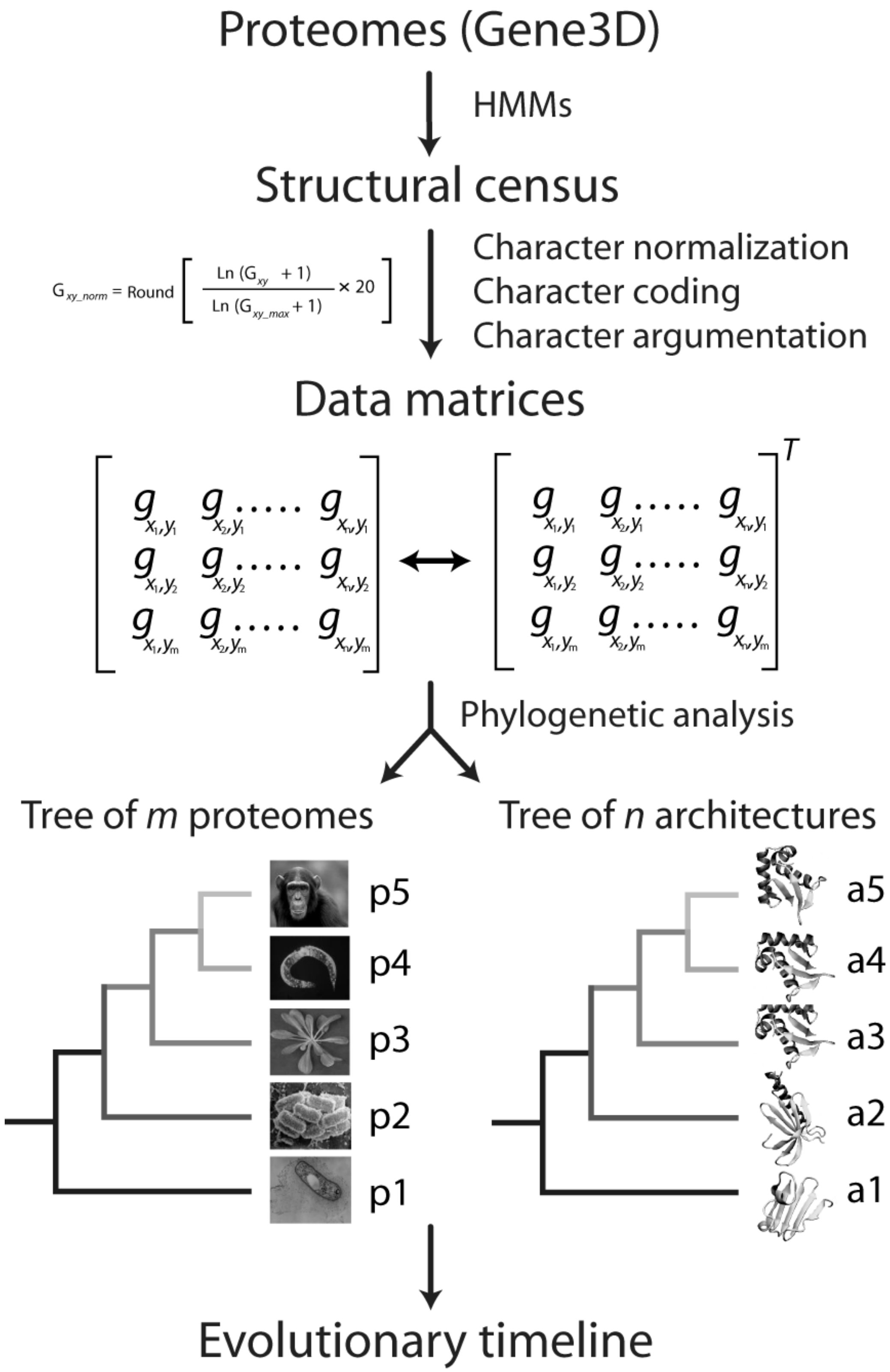

Figure S1 A methodological flowchart illustrating the reconstruction of phylogenies of proteomes and protein architectures using protein domain census data.
