# Supplementary material for: Origin and Evolution of Protein Fold Designs Inferred from Phylogenomic Analysis of CATH Domain Structures in Proteomes
Source: PLoS Comput Biol. 2013 Mar 28;9(3):e1003009. doi: 10.1371/journal.pcbi.1003009 (PMC3610613; doi:10.1371/journal.pcbi.1003009)
Supplement: Protocol S1 — By hand example of protein domain data normalization and encoding. The objective is to build a phylogenomic matrix for the reconstruction of a tree of domain structures. (PDF) [file pcbi.1003009.s003.pdf]

**Protocol S1. By hand example of protein domain data normalization and encoding.** The objective is to build a phylogenomic matrix for the reconstruction of a tree of domain structures.

1. Generate a matrix of domain abundances in genomes (Table 1).

**Table 1.** Structural census in genomes

| | **Gnom-1** | **Gnom-2** | **Gnom-3** | **Gnom-4** | **Gnom-5** | **Gnom-6** | $G_{max\_row}$ |
|---|---|---|---|---|---|---|---|
| Domain 1 | 2127 | 200 | 5000 | 2689 | 2411 | 5 | 5000 |
| Domain 2 | 0 | 1200 | 300 | 553 | 20 | 1000 | 1200 |
| Domain 3 | 9 | 11 | 8 | 2 | 2 | 2 | 11 |

Gnom-1,…Gnom-6 represent genomes.

2. Normalize abundances using the following character normalization formula:

$$G_{xy\_norm} = \text{Round}\left[\frac{\ln(G_{xy}+1)}{\ln(G_{xy\_max}+1)} \times 20\right]$$

where $G_{xy}$ is the frequency of domain x in Gnom-y, and $G_{xy\_max} = G_{max\_row}$ , given that the objective is normalization of a census of domains for building a tree of domains.

Abundances are normalized for genome sizes and are expressed on a 0-20 scale (Table 2); values are now character states.

**Table 2.** Character normalization and scaling

| | **Gnom1** | **Gnom2** | **Gnom3** | **Gnom4** | **Gnom5** | **Gnom6** |
|---|---|---|---|---|---|---|
| Domain 1 | 18 | 12 | 20 | 19 | 18 | 4 |
| Domain 2 | 0 | 20 | 16 | 18 | 9 | 19 |
| Domain 3 | 19 | 20 | 18 | 9 | 9 | 9 |

3. Character states are encoded as linearly ordered multistate phylogenetic characters using an alphanumeric format of numbers 0-9 and letters A-K, which are compatible with PAUP* (Table 3).

**Table 3.** Character encoding

| | **Gnom1** | **Gnom2** | **Gnom3** | **Gnom4** | **Gnom5** | **Gnom6** |
|---|---|---|---|---|---|---|
| Domain 1 | I | C | K | J | I | 4 |
| Domain 2 | 0 | K | G | I | 9 | J |
| Domain 3 | J | K | I | 9 | 9 | 9 |
