# Supplementary material for: Origin and Evolution of Protein Fold Designs Inferred from Phylogenomic Analysis of CATH Domain Structures in Proteomes
Source: PLoS Comput Biol. 2013 Mar 28;9(3):e1003009. doi: 10.1371/journal.pcbi.1003009 (PMC3610613; doi:10.1371/journal.pcbi.1003009)
Supplement: Table S1 — List of 15 most ancient and popular Hs. (PDF) [file pcbi.1003009.s004.pdf]

Table S1 List of 15 most ancient and popular Hs.

Index	CATH domains	Keyword	nd value	f value
**1**	3.40.50.300	P-loop containing nucleotide triphosphate hydrolases	0	1
**2**	3.40.50.150	Vaccinia Virus protein VP39	0.009803922	1
**3**	3.40.50.720	NAD(P)-binding Rossmann-like Domain	0.009803922	1
**4**	3.50.50.60	FAD/NAD(P)-binding domain	0.009803922	1
**5**	3.40.50.620	Tyrosyl-Transfer RNA Synthetase , subunit E, domain 1	0.014705882	1
**6**	3.20.20.70	Aldolase class I	0.019607843	0.9959184
**7**	3.90.550.10	Spore Coat Polysaccharide Biosynthesis Protein SpsA; Chain A	0.024509804	0.9918367
**8**	3.40.50.1000	Rossmann fold	0.034313725	0.9979592
**9**	2.40.50.140	Nucleic acid-binding proteins	0.039215686	1
**10**	3.20.20.140	Metal-dependent hydrolases	0.039215686	0.9979592
**11**	1.10.10.10	winged helix repressor DNA binding domain	0.044117647	1
**12**	3.40.640.10	Type I PLP-dependent aspartate aminotransferase-like (Major domain)	0.044117647	0.9959184
**13**	3.90.1150.10	Aspartate Aminotransferase, domain 1	0.044117647	0.9959184
**14**	1.10.8.60	Helicase, Ruva Protein; domain 3	0.049019608	1
**15**	2.40.30.10	Translation factors	0.049019608	1
