# Supplementary material for: Origin and Evolution of Protein Fold Designs Inferred from Phylogenomic Analysis of CATH Domain Structures in Proteomes
Source: PLoS Comput Biol. 2013 Mar 28;9(3):e1003009. doi: 10.1371/journal.pcbi.1003009 (PMC3610613; doi:10.1371/journal.pcbi.1003009)
Supplement: Table S2 — List of 492 organisms with their genome id and genome names. (PDF) [file pcbi.1003009.s005.pdf]

Table S2 List of 492 organisms with their genome id and genome names. A, B and E letters at the end of genome names refers to superkingdoms Archaea, Bacteria and Eukarya, respectively.

Genome ID	*Genome Name*
00	Campylobacter hominis_B
01	Polaromonas naphthalenivorans_B
02	Metallosphaera sedula_A
03	Clostridium beijerinckii_B
04	Borrelia afzelii_B
09	Pelotomaculum thermopropionicum_B
0C	Anaplasma marginale_B
0G	Sulfolobus islandicus_A
0K	Escherichia coli_B
0L	Streptococcus suis_B
0N	Laribacter hongkongensis_B
0P	Gluconacetobacter diazotrophicus_B
0R	Atopobium parvulum_B
0S	Streptococcus equi_B
0V	Desulfovibrio desulfuricans_B
0X	Thermococcus sibiricus_A
0Z	Clostridium cellulolyticum_B
11	Methanosaeta thermophila_A
12	Clostridium novyi_B
13	Lactobacillus gasseri_B
14	Methylibium petroleiphilum_B
16	Clostridium kluyveri_B
17	Rhizobium leguminosarum_B
18	Saccharopolyspora erythraea_B
1B	Deinococcus deserti_B
1E	Brucella melitensis_B
1H	Anaerococcus prevotii_B
1N	Clostridium botulinum_B
1O	Eggerthella lenta_B
1P	Bacillus cereus_B
1Q	Saccharomonospora viridis_B
1R	Mycobacterium tuberculosis_B
1S	Acetobacter pasteurianus_B
1U	Streptococcus pneumoniae_B
1V	Macrococcus caseolyticus_B
1W	Methylotenera mobilis_B
1X	Helicobacter pylori_B

Table S2 (contd.)

Genome ID	*Genome Name*
1Z	Actinosynnema mirum_B
23	Methanoculleus marisnigri_A
27	Paracoccus denitrificans_B
28	Mycobacterium vanbaalenii_B
2A	Klebsiella pneumoniae_B
2B	Variovorax paradoxus_B
2D	Dyadobacter fermentans_B
2E	Kosmotoga olearia_B
2G	Kytococcus sedentarius_B
2K	Desulfohalobium retbaense_B
2L	Acidobacterium capsulatum_B
2O	Teredinibacter turnerae_B
2P	Desulfotomaculum acetoxidans_B
2Q	Micrococcus luteus_B
2V	Nakamurella multipartita_B
2X	Jonesia denitrificans_B
2Y	Burkholderia glumae_B
2Z	Ralstonia pickettii_B
30	Burkholderia vietnamiensis_B
32	Clavibacter michiganensis_B
33	Aeromonas hydrophila_B
34	Pyrobaculum islandicum_A
35	Campylobacter fetus_B
37	Cytophaga hutchinsonii_B
3B	Caulobacter crescentus_B
3C	Burkholderia pseudomallei_B
3E	Rhodococcus opacus_B
3G	Brachybacterium faecium_B
3I	Dickeya zeae_B
3J	Brachyspira hyodysenteriae_B
3L	Aggregatibacter aphrophilus_B
3M	Tolumonas auensis_B
3N	Eubacterium eligens_B
3P	Methanocaldococcus fervens_A
3Q	Rhodobacter sphaeroides_B
3R	Halorhabdus utahensis_A
3W	Desulfobacterium autotrophicum_B
3Y	Pedobacter heparinus_B
40	Ochrobactrum anthropi_B
44	Aeromonas salmonicida_B
45	Mycoplasma agalactiae_B

Table S2 (contd.)

Genome ID	*Genome Name*
49	Lactobacillus reuteri_B
4A	Alicyclobacillus acidocaldarius_B
4B	Halorubrum lacusprofundi_A
4C	Slackia heliotrinireducens_B
4D	Catenulispora acidiphila_B
4H	Kangiella koreensis_B
4I	Campylobacter lari_B
4L	Lactobacillus plantarum_B
4M	Thermomicrobium roseum_B
4T	Pseudomonas fluorescens_B
4U	Listeria monocytogenes_B
4V	Flavobacteriaceae bacterium_B
4X	Halomicrobium mukohataei_A
4Y	Mycobacterium leprae_B
52	Lactobacillus brevis_B
54	Hyphomonas neptunium_B
56	Arthrobacter aurescens_B
57	Methanococcus vannielii_A
58	Kineococcus radiotolerans_B
5B	Salmonella enterica_B
5D	Halothermothrix orenii_B
5E	Anaerocellum thermophilum_B
5O	Vibrio cholerae_B
5R	Lactobacillus rhamnosus_B
5S	Staphylococcus carnosus_B
5U	Beutenbergia cavernae_B
5V	Thermococcus gammatolerans_A
5Y	Chitinophaga pinensis_B
60	Sorangium cellulosum_B
61	Salinispora arenicola_B
62	Bacillus weihenstephanensis_B
63	Ignicoccus hospitalis_A
65	Vibrio harveyi_B
68	Alkaliphilus oremlandii_B
69	Cronobacter sakazakii_B
6A	Methylobacterium extorquens_B
6B	Gemmatimonas aurantiaca_B
6C	Nautilia profundicola_B
6D	Desulfomicrobium baculatum_B
6E	Arthrobacter chlorophenolicus_B
6H	Mycoplasma hominis_B

Table S2 (contd.)

Genome ID	*Genome Name*
6Q	Rothia mucilaginosa_B
6R	Xanthomonas albilineans_B
6T	Staphylococcus lugdunensis_B
6U	Lactococcus lactis_B
6W	Listeria seeligeri_B
74	Rickettsia akari_B
75	Azorhizobium caulinodans_B
76	Burkholderia multivorans_B
77	Roseiflexus castenholzii_B
79	Lactobacillus helveticus_B
7D	Streptococcus gallolyticus_B
7E	Streptomyces scabiei_B
7F	Geodermatophilus obscurus_B
7I	Xylanimonas cellulosilytica_B
7N	Deferribacter desulfuricans_B
7O	Thermocrinis albus_B
7R	Erwinia pyrifoliae_B
7S	Aggregatibacter actinomycetemcomitans_B
7V	Clostridium difficile_B
7W	Archaeoglobus profundus_A
7Z	Sphaerobacter thermophilus_B
81	Acaryochloris marina_B
82	Thermoanaerobacter pseudethanolicus_B
83	Leptospira biflexa_B
84	Microcystis aeruginosa_B
88	Shewanella woodyi_B
8C	Lactobacillus johnsonii_B
8G	Gordonia bronchialis_B
8H	Erwinia amylovora_B
8I	Zymomonas mobilis_B
8J	Salinibacter ruber_B
8L	Veillonella parvula_B
8M	Edwardsiella tarda_B
8P	Sebaldella termitidis_B
8Q	Legionella longbeachae_B
8T	Fibrobacter succinogenes_B
8U	Streptosporangium roseum_B
90	Burkholderia phymatum_B
94	Elusimicrobium minutum_B
98	Kocuria rhizophila_B
9A	Staphylococcus aureus_B

Table S2 (contd.)

Genome ID	*Genome Name*
9C	Thermanaerovibrio acidaminovorans_B
9H	Stackebrandtia nassauensis_B
9L	Hydrogenobacter thermophilus_B
9O	Aciduliprofundum boonei_A
9Q	Methanobrevibacter ruminantium_A
9W	alpha proteobacterium_B
9Y	Pantoea ananatis_B
9Z	Clostridiales genomosp._B
C1	Callithrix jacchus_E
CD	Candida dubliniensis_E
PC	Penicillium chrysogenum_E
SS	Sus scrofa_E
TV	Trichophyton verrucosum_E
a5	Aspergillus niger_E
a7	Aspergillus clavatus_E
a8	Aspergillus oryzae_E
aA	Pirellula staleyi_B
aE	Haliangium ochraceum_B
aG	Haloferax volcanii_A
aL	Meiothermus ruber_B
aN	Bacillus pseudofirmus_B
aP	Streptococcus mitis_B
aQ	Ferroglobus placidus_A
aT	Chlamydia trachomatis_B
au	Agrobacterium tumefaciens_B
av	Mycobacterium avium_B
ax	Aedes aegypti_E
az	Aromatoleum aromaticum_B
b1	Baumannia cicadellinicola_B
b2	Bacillus anthracis_B
b3	Brucella abortus_B
b4	Burkholderia xenovorans_B
b5	Burkholderia thailandensis_B
b6	Burkholderia cenocepacia_B
bb	Borrelia burgdorferi_B
be	Bordetella pertussis_B
bh	Bacillus halodurans_B
bi	Burkholderia mallei_B
bj	Bradyrhizobium japonicum_B
bl	Bifidobacterium longum_B
bn	Buchnera aphidicola_B

Table S2 (contd.)

Genome ID	*Genome Name*
bo	Bordetella bronchiseptica_B
bp	Bordetella parapertussis_B
bq	Bartonella quintana_B
br	Brucella suis_B
bs	Bacillus subtilis_B
bt	Bacteroides thetaiotaomicron_B
bv	Bos taurus_E
c0	Ciona savignyi_E
c2	Chlamydophila pneumoniae_B
c3	Corynebacterium glutamicum_B
c4	Chlamydophila abortus_B
c5	Chlorobium chlorochromatii_B
c6	Chlamydophila felis_B
c8	Chromohalobacter salexigens_B
c9	Carboxydothermus hydrogenoformans_B
ca	Clostridium acetobutylicum_B
cf	Cryptococcus neoformans_E
ch	Chlorobium tepidum_B
cl	Caenorhabditis elegans_E
cm	Chlamydia muridarum_B
co	Corynebacterium efficiens_B
cv	Cryptosporidium parvum_E
cw	Caenorhabditis briggsae_E
d0	Saccharophagus degradans_B
d1	Shigella dysenteriae_B
d2	Desulfitobacterium hafniense_B
d4	Deinococcus geothermalis_B
d5	Dasypus novemcinctus_E
da	Danio rerio_E
dd	Drosophila melanogaster_E
dj	Dechloromonas aromatica_B
do	Drosophila pseudoobscura_E
dp	Desulfotalea psychrophila_B
dr	Deinococcus radiodurans_B
dt	Dictyostelium discoideum_E
dv	Desulfovibrio vulgaris_B
e9	Stenotrophomonas maltophilia_B
ee	Echinops telfairi_E
ef	Enterococcus faecalis_B
eg	Ehrlichia chaffeensis_B
eh	Ehrlichia ruminantium_B

Table S2 (contd.)

Genome ID	*Genome Name*
ej	Erwinia tasmaniensis_B
ek	Erinaceus europaeus_E
el	Ehrlichia canis_B
em	Leishmania major_E
ep	Staphylococcus epidermidis_B
eq	Equus caballus_E
er	Pectobacterium atrosepticum_B
et	Dehalococcoides ethenogenes_B
eu	Encephalitozoon cuniculi_E
ev	Chlorobaculum parvum_B
ey	Erythrobacter litoralis_B
f7	Borrelia duttonii_B
fb	Plasmodium berghei_E
fd	Desulfurococcus kamchatkensis_A
fe	Felis catus_E
fj	Methylobacterium populi_B
fl	Bacteroides fragilis_B
fn	Fusobacterium nucleatum_B
fp	Chlorobium limicola_B
fs	Shigella flexneri_B
ft	Francisella tularensis_B
fu	Methanosarcina barkeri_A
fw	Plasmodium knowlesi_E
fy	Plasmodium chabaudi_E
g5	Bifidobacterium animalis_B
g7	Borrelia recurrentis_B
ga	Borrelia garinii_B
gb	Spermophilus tridecemlineatus_E
gc	Gasterosteus aculeatus_E
gf	Giardia lamblia_E
gg	Gallus gallus_E
gi	Aspergillus terreus_E
gk	Geobacillus kaustophilus_B
gl	Candida glabrata_E
gm	Geobacter metallireducens_B
go	Ashbya gossypii_E
gq	Aspergillus flavus_E
gs	Geobacter sulfurreducens_B
gt	Guillardia theta_E
gu	Cavia porcellus_E
gv	Gloeobacter violaceus_B

Table S2 (contd.)

Genome ID	***Genome Name***
gx	Gorilla gorilla_E
h3	Coprothermobacter proteolyticus_B
h5	Shewanella piezotolerans_B
h6	Thermococcus onnurineus_A
ha	Pseudoalteromonas haloplanktis_B
hc	Hahella chejuensis_B
hd	Haemophilus ducreyi_B
he	Photobacterium profundum_B
hg	Chaetomium globosum_E
hh	Helicobacter hepaticus_B
hi	Haemophilus influenzae_B
hl	Helicobacter acinonychis_B
hm	Haloarcula marismortui_A
ho	Bartonella henselae_B
hs	Homo sapiens_E
hw	Shewanella denitrificans_B
ib	Leishmania braziliensis_E
ih	Tarsius syrichta_E
il	Idiomarina loihiensis_B
io	Microcebus murinus_E
ir	Paramecium tetraurelia_E
is	Ciona intestinalis_E
ix	Dictyoglomus turgidum_B
j0	Natranaerobius thermophilus_B
j5	Methylobacterium chloromethanicum_B
j6	Anoxybacillus flavithermus_B
jb	Chloroflexus aggregans_B
jj	Oligotropha carboxidovorans_B
jk	Corynebacterium jeikeium_B
jn	Proteus mirabilis_B
jt	Thermosipho africanus_B
jw	Geobacter bemidjiensis_B
jx	Methanosphaerula palustris_A
k1	Aliivibrio salmonicida_B
k3	Dictyoglomus thermophilum_B
k7	Thermodesulfovibrio yellowstonii_B
kb	Alteromonas macleodii_B
kd	Escherichia fergusonii_B
ke	Methylocella silvestris_B
kf	Ureaplasma urealyticum_B
kg	Rhodospirillum centenum_B

Table S2 (contd.)

Genome ID	*Genome Name*
kj	Acidithiobacillus ferrooxidans_B
kk	Phenylobacterium zucineum_B
kl	Kluyveromyces lactis_E
km	Haemophilus parasuis_B
kn	Vibrio splendidus_B
ko	Bacillus thuringiensis_B
ku	Burkholderia phytofirmans_B
ld	Lactobacillus delbrueckii_B
lf	Bacillus licheniformis_B
lh	Leishmania infantum_E
li	Listeria innocua_B
lk	Loxodonta africana_E
ln	Lawsonia intracellularis_B
lr	Leptospira interrogans_B
ls	Lactobacillus sakei_B
lt	Lactobacillus acidophilus_B
lu	Myotis lucifugus_E
lv	Lactobacillus salivarius_B
lw	Colwellia psychrerythraea_B
lx	Leifsonia xyli_B
ly	Lodderomyces elongisporus_E
m0	Mycoplasma mobile_B
m2	Methanococcus maripaludis_A
m3	Mannheimia succiniciproducens_B
m4	Methanosphaera stadtmanae_A
m5	Magnetospirillum magneticum_B
m6	Methanospirillum hungatei_A
m7	Methylobacillus flagellatus_B
m8	Moorella thermoacetica_B
m9	Methanococcoides burtonii_A
ma	Methanosarcina acetivorans_A
mc	Mycobacterium bovis_B
md	Methanothermobacter thermautotrophicus_A
me	Mycoplasma penetrans_B
mf	Mesoplasma florum_B
mg	Mycoplasma genitalium_B
mj	Methanocaldococcus jannaschii_A
mk	Mesorhizobium loti_B
mm	Mus musculus_E
mn	Methanopyrus kandleri_A
mp	Mycoplasma pneumoniae_B

Table S2 (contd.)

Genome ID	*Genome Name*
mq	Mycoplasma pulmonis_B
mt	Methylococcus capsulatus_B
my	Mycoplasma gallisepticum_B
mz	Methanosarcina mazei_A
na	Nanoarchaeum equitans_A
nb	Nitrobacter hamburgensis_B
ne	Nitrosomonas europaea_B
nf	Nocardia farcinica_B
nh	Neosartorya fischeri_E
ni	Neisseria gonorrhoeae_B
nl	Nitrosospira multiformis_B
nn	Neisseria meningitidis_B
np	Natronomonas pharaonis_A
nr	Nitrosococcus oceani_B
ns	Neurospora crassa_E
nt	Thiobacillus denitrificans_B
nu	Theileria annulata_E
nv	Novosphingobium aromaticivorans_B
nw	Nematostella vectensis_E
o0	Babesia bovis_E
ob	Otolemur garnettii_E
of	Pongo pygmaeus_E
oh	Ornithorhynchus anatinus_E
oi	Oceanobacillus iheyensis_B
ok	Oryctolagus cuniculus_E
ol	Oryzias latipes_E
on	Shigella sonnei_B
op	Monodelphis domestica_E
oq	Ochotona princeps_E
os	Oryza sativa_E
ou	Ostreococcus tauri_E
ov	Monosiga brevicollis_E
ox	Gluconobacter oxydans_B
oz	Ostreococcus lucimarinus_E
p1	Prochlorococcus marinus_B
p3	Picrophilus torridus_A
p8	Pelobacter carbinolicus_B
pa	Pseudomonas aeruginosa_B
pb	Pyrococcus abyssi_A
pd	Photorhabdus luminescens_B
pg	Porphyromonas gingivalis_B

Table S2 (contd.)

Genome ID	*Genome Name*
ph	Pyrococcus horikoshii_A
pj	Pseudomonas syringae_B
pl	Plasmodium falciparum_E
pq	Pseudomonas entomophila_B
ps	Pseudomonas putida_B
pu	Pyrococcus furiosus_A
pv	Theileria parva_E
px	Pseudoalteromonas atlantica_B
py	Plasmodium yoelii_E
pz	Psychrobacter cryohalolentis_B
r3	Rickettsia bellii_B
rb	Rhodospirillum rubrum_B
rc	Rickettsia conorii_B
rd	Rhodopseudomonas palustris_B
rf	Rickettsia felis_B
ri	Psychrobacter arcticus_B
rl	Ralstonia eutropha_B
rm	Cryptosporidium hominis_E
rn	Rattus norvegicus_E
rp	Rickettsia prowazekii_B
rs	Ralstonia solanacearum_B
rt	Rickettsia typhi_B
ru	Macaca mulatta_E
rw	Rubrobacter xylanophilus_B
rx	Rhodoferax ferrireducens_B
rz	Rhizobium etli_B
s5	Streptococcus agalactiae_B
s6	Streptococcus mutans_B
s9	Streptomyces avermitilis_B
sb	Symbiobacterium thermophilum_B
sc	Saccharomyces cerevisiae_E
sd	Streptococcus thermophilus_B
sf	Streptomyces coelicolor_B
sg	Ruegeria pomeroyi_B
sm	Sinorhizobium meliloti_B
sq	Shigella boydii_B
sv	Sulfolobus tokodaii_A
t0	Thermus thermophilus_B
ta	Thermoplasma acidophilum_A
tc	Thiomicrospira crunogena_B
td	Treponema denticola_B

Table S2 (contd.)

Genome ID	*Genome Name*
te	Clostridium tetani_B
tf	Thermobifida fusca_B
ti	Sulfurimonas denitrificans_B
tk	Thermococcus kodakarensis_A
tn	Tetraodon nigroviridis_E
to	Takifugu rubripes_E
tw	Tropheryma whipplei_B
tz	Tupaia belangeri_E
ue	Toxoplasma gondii_E
um	Ustilago maydis_E
ut	Tursiops truncatus_E
uu	Ureaplasma parvum_B
uz	Trypanosoma cruzi_E
va	Anabaena variabilis_B
vb	Vibrio vulnificus_B
vf	Vibrio fischeri_B
vi	Chromobacterium violaceum_B
vn	Procavia capensis_E
vp	Vibrio parahaemolyticus_B
vr	Pteropus vampyrus_E
vw	Vanderwaltozyma polyspora_E
vx	Plasmodium vivax_E
wb	Wigglesworthia glossinidia_B
wi	Nitrobacter winogradskyi_B
ws	Wolinella succinogenes_B
x2	Streptococcus pyogenes_B
x3	Synechococcus elongatus_B
x4	Bacillus clausii_B
x7	Legionella pneumophila_B
x9	Mycoplasma hyopneumoniae_B
xc	Xanthomonas axonopodis_B
xd	Xanthomonas campestris_B
xf	Xylella fastidiosa_B
xo	Xanthomonas oryzae_B
xp	Pan troglodytes_E
xr	Sorex araneus_E
yc	Mycoplasma synoviae_B
yi	Mycoplasma capricolum_B
yl	Yarrowia lipolytica_E
yp	Yersinia pestis_B
yr	Yersinia pseudotuberculosis_B

Table S2 (contd.)

Genome ID	*Genome Name*
yt	Syntrophus aciditrophicus_B
za	Sulfolobus acidocaldarius_A
zh	Staphylococcus haemolyticus_B
zt	Staphylococcus saprophyticus_B
