# Supplementary material for: Origin and Evolution of Protein Fold Designs Inferred from Phylogenomic Analysis of CATH Domain Structures in Proteomes
Source: PLoS Comput Biol. 2013 Mar 28;9(3):e1003009. doi: 10.1371/journal.pcbi.1003009 (PMC3610613; doi:10.1371/journal.pcbi.1003009)
Supplement: Table S3 — List of 295 FL proteomes used to reconstruct proteome trees. (PDF) [file pcbi.1003009.s006.pdf]

Table S3 List of 295 FL proteomes used to reconstruct proteome trees.

Genome ID	*Phylum*	*Superkingdom*	*NCBI Taxonomy ID*
cf	Fungi	Eukaryota	214684
hg	Fungi	Eukaryota	306901
ns	Fungi	Eukaryota	367110
nh	Fungi	Eukaryota	331117
gi	Fungi	Eukaryota	341663
a8	Fungi	Eukaryota	5062
a5	Fungi	Eukaryota	425011
gq	Fungi	Eukaryota	332952
a7	Fungi	Eukaryota	344612
ly	Fungi	Eukaryota	379508
yl	Fungi	Eukaryota	284591
vw	Fungi	Eukaryota	436907
gl	Fungi	Eukaryota	284593
go	Fungi	Eukaryota	284811
sc	Fungi	Eukaryota	4932
kl	Fungi	Eukaryota	284590
hs	Metazoa	Eukaryota	9606
xp	Metazoa	Eukaryota	9598
gx	Metazoa	Eukaryota	9593
of	Metazoa	Eukaryota	9600
ru	Metazoa	Eukaryota	9544
C1	Metazoa	Eukaryota	9483
ob	Metazoa	Eukaryota	30611
io	Metazoa	Eukaryota	30608
ih	Metazoa	Eukaryota	9478
rn	Metazoa	Eukaryota	10116
mm	Metazoa	Eukaryota	10090
gb	Metazoa	Eukaryota	43179
gu	Metazoa	Eukaryota	10141
ok	Metazoa	Eukaryota	9986
oq	Metazoa	Eukaryota	9978
tz	Metazoa	Eukaryota	37347
SS	Metazoa	Eukaryota	9823
bv	Metazoa	Eukaryota	9913
ut	Metazoa	Eukaryota	9739
fe	Metazoa	Eukaryota	9685
eq	Metazoa	Eukaryota	9796
lu	Metazoa	Eukaryota	59463
vr	Metazoa	Eukaryota	132908

Table S3 (contd.)

Genome ID	*Phylum*	*Superkingdom*	*NCBI Taxonomy ID*
xr	Metazoa	Eukaryota	42254
ek	Metazoa	Eukaryota	9365
vn	Metazoa	Eukaryota	9813
lk	Metazoa	Eukaryota	9785
ee	Metazoa	Eukaryota	9371
d5	Metazoa	Eukaryota	9361
op	Metazoa	Eukaryota	13616
oh	Metazoa	Eukaryota	9258
gg	Metazoa	Eukaryota	9031
da	Metazoa	Eukaryota	7955
gc	Metazoa	Eukaryota	69293
ol	Metazoa	Eukaryota	8090
tn	Metazoa	Eukaryota	99883
to	Metazoa	Eukaryota	31033
c0	Metazoa	Eukaryota	51511
is	Metazoa	Eukaryota	7719
do	Metazoa	Eukaryota	46245
dd	Metazoa	Eukaryota	7227
ax	Metazoa	Eukaryota	7159
cl	Metazoa	Eukaryota	6239
cw	Metazoa	Eukaryota	6238
nw	Metazoa	Eukaryota	45351
ir	Protista	Eukaryota	5888
os	Plantae	Eukaryota	39947
oz	Plantae	Eukaryota	436017
ou	Plantae	Eukaryota	70448
2L	Acidobacteria	Bacteria	240015
g5	Actinobacteria	Bacteria	580050
58	Actinobacteria	Bacteria	266940
4D	Actinobacteria	Bacteria	479433
9H	Actinobacteria	Bacteria	446470
2V	Actinobacteria	Bacteria	479431
7F	Actinobacteria	Bacteria	526225
tf	Actinobacteria	Bacteria	269800
8U	Actinobacteria	Bacteria	479432
s9	Actinobacteria	Bacteria	227882
sf	Actinobacteria	Bacteria	100226
18	Actinobacteria	Bacteria	405948
61	Actinobacteria	Bacteria	391037
3E	Actinobacteria	Bacteria	632772
nf	Actinobacteria	Bacteria	247156

Table S3 (contd.)

Genome ID	*Phylum*	*Superkingdom*	*NCBI Taxonomy ID*
av	Actinobacteria	Bacteria	262316
28	Actinobacteria	Bacteria	350058
co	Actinobacteria	Bacteria	196164
c3	Actinobacteria	Bacteria	196627
5U	Actinobacteria	Bacteria	471853
2X	Actinobacteria	Bacteria	471856
3G	Actinobacteria	Bacteria	446465
6E	Actinobacteria	Bacteria	452863
56	Actinobacteria	Bacteria	290340
2Q	Actinobacteria	Bacteria	596312
4C	Actinobacteria	Bacteria	471855
0R	Actinobacteria	Bacteria	521095
rw	Actinobacteria	Bacteria	266117
7O	Aquificae	Bacteria	638303
9L	Aquificae	Bacteria	608538
37	Bacteroidetes	Bacteria	269798
8J	Bacteroidetes	Bacteria	309807
5Y	Bacteroidetes	Bacteria	485918
3Y	Bacteroidetes	Bacteria	485917
4V	Bacteroidetes	Bacteria	531844
fp	Chlorobi	Bacteria	290315
ev	Chlorobi	Bacteria	517417
ch	Chlorobi	Bacteria	194439
et	Chloroflexi	Bacteria	243164
4M	Chloroflexi	Bacteria	309801
7Z	Chloroflexi	Bacteria	479434
77	Chloroflexi	Bacteria	383372
jb	Chloroflexi	Bacteria	326427
gv	Cyanobacteria	Bacteria	251221
81	Cyanobacteria	Bacteria	329726
p1	Cyanobacteria	Bacteria	59922
va	Cyanobacteria	Bacteria	240292
84	Cyanobacteria	Bacteria	449447
7N	Deferribacteres	Bacteria	639282
1B	Deinococcus-Thermus	Bacteria	546414
d4	Deinococcus-Thermus	Bacteria	319795
dr	Deinococcus-Thermus	Bacteria	243230
aL	Deinococcus-Thermus	Bacteria	504728
t0	Deinococcus-Thermus	Bacteria	262724
ix	Dictyoglomi	Bacteria	515635
k3	Dictyoglomi	Bacteria	309799

Table S3 (contd.)

Genome ID	*Phylum*	*Superkingdom*	*NCBI Taxonomy ID*
8T	Fibrobacteres	Bacteria	59374
j0	Firmicutes	Bacteria	457570
9	Firmicutes	Bacteria	370438
d2	Firmicutes	Bacteria	138119
2P	Firmicutes	Bacteria	485916
68	Firmicutes	Bacteria	350688
12	Firmicutes	Bacteria	386415
16	Firmicutes	Bacteria	431943
0Z	Firmicutes	Bacteria	394503
3	Firmicutes	Bacteria	290402
7V	Firmicutes	Bacteria	645463
1N	Firmicutes	Bacteria	592027
ca	Firmicutes	Bacteria	272562
5E	Firmicutes	Bacteria	521460
h3	Firmicutes	Bacteria	309798
c9	Firmicutes	Bacteria	246194
m8	Firmicutes	Bacteria	264732
82	Firmicutes	Bacteria	340099
5D	Firmicutes	Bacteria	373903
ls	Firmicutes	Bacteria	314315
4L	Firmicutes	Bacteria	220668
79	Firmicutes	Bacteria	405566
ld	Firmicutes	Bacteria	390333
52	Firmicutes	Bacteria	387344
6U	Firmicutes	Bacteria	272623
4A	Firmicutes	Bacteria	543302
li	Firmicutes	Bacteria	272626
6W	Firmicutes	Bacteria	683837
oi	Firmicutes	Bacteria	221109
j6	Firmicutes	Bacteria	491915
gk	Firmicutes	Bacteria	235909
bs	Firmicutes	Bacteria	224308
lf	Firmicutes	Bacteria	279010
bh	Firmicutes	Bacteria	272558
62	Firmicutes	Bacteria	315730
ko	Firmicutes	Bacteria	527024
1P	Firmicutes	Bacteria	526976
b2	Firmicutes	Bacteria	405536
aN	Firmicutes	Bacteria	398511
x4	Firmicutes	Bacteria	66692
1V	Firmicutes	Bacteria	458233

Table S3 (contd.)

Genome ID	*Phylum*	*Superkingdom*	*NCBI Taxonomy ID*
5S	Firmicutes	Bacteria	396513
6B	Gemmatimonadetes	Bacteria	379066
k7	Nitrospirae	Bacteria	289376
aA	Planctomycetes	Bacteria	530564
6C	Proteobacteria	Bacteria	598659
ti	Proteobacteria	Bacteria	326298
yt	Proteobacteria	Bacteria	56780
dp	Proteobacteria	Bacteria	177439
3W	Proteobacteria	Bacteria	177437
2K	Proteobacteria	Bacteria	485915
6D	Proteobacteria	Bacteria	525897
dv	Proteobacteria	Bacteria	883
0V	Proteobacteria	Bacteria	525146
p8	Proteobacteria	Bacteria	338963
jw	Proteobacteria	Bacteria	404380
gs	Proteobacteria	Bacteria	243231
gm	Proteobacteria	Bacteria	269799
60	Proteobacteria	Bacteria	448385
az	Proteobacteria	Bacteria	76114
dj	Proteobacteria	Bacteria	159087
vi	Proteobacteria	Bacteria	243365
1W	Proteobacteria	Bacteria	583345
m7	Proteobacteria	Bacteria	265072
nt	Proteobacteria	Bacteria	292415
14	Proteobacteria	Bacteria	420662
rl	Proteobacteria	Bacteria	381666
rs	Proteobacteria	Bacteria	305
b5	Proteobacteria	Bacteria	271848
3C	Proteobacteria	Bacteria	320373
b6	Proteobacteria	Bacteria	331271
30	Proteobacteria	Bacteria	269482
b4	Proteobacteria	Bacteria	266265
rx	Proteobacteria	Bacteria	338969
1	Proteobacteria	Bacteria	365044
2B	Proteobacteria	Bacteria	543728
nl	Proteobacteria	Bacteria	323848
ne	Proteobacteria	Bacteria	228410
3B	Proteobacteria	Bacteria	565050
ey	Proteobacteria	Bacteria	314225
nv	Proteobacteria	Bacteria	279238
8I	Proteobacteria	Bacteria	264203

Table S3 (contd.)

Genome ID	*Phylum*	*Superkingdom*	*NCBI Taxonomy ID*
54	Proteobacteria	Bacteria	228405
3Q	Proteobacteria	Bacteria	272943
27	Proteobacteria	Bacteria	318586
m5	Proteobacteria	Bacteria	342108
kg	Proteobacteria	Bacteria	414684
rb	Proteobacteria	Bacteria	269796
0P	Proteobacteria	Bacteria	272568
ox	Proteobacteria	Bacteria	290633
1S	Proteobacteria	Bacteria	634457
j5	Proteobacteria	Bacteria	440085
6A	Proteobacteria	Bacteria	661410
fj	Proteobacteria	Bacteria	441620
40	Proteobacteria	Bacteria	439375
ke	Proteobacteria	Bacteria	395965
jj	Proteobacteria	Bacteria	504832
rd	Proteobacteria	Bacteria	316055
wi	Proteobacteria	Bacteria	323098
nb	Proteobacteria	Bacteria	323097
kj	Proteobacteria	Bacteria	243159
3M	Proteobacteria	Bacteria	595494
33	Proteobacteria	Bacteria	380703
vp	Proteobacteria	Bacteria	419109
65	Proteobacteria	Bacteria	410291
vb	Proteobacteria	Bacteria	216895
5O	Proteobacteria	Bacteria	417398
he	Proteobacteria	Bacteria	74109
il	Proteobacteria	Bacteria	283942
h5	Proteobacteria	Bacteria	225849
hw	Proteobacteria	Bacteria	318161
88	Proteobacteria	Bacteria	392500
lw	Proteobacteria	Bacteria	167879
px	Proteobacteria	Bacteria	342610
ha	Proteobacteria	Bacteria	326442
d0	Proteobacteria	Bacteria	203122
kb	Proteobacteria	Bacteria	314275
hc	Proteobacteria	Bacteria	349521
4H	Proteobacteria	Bacteria	523791
c8	Proteobacteria	Bacteria	290398
mt	Proteobacteria	Bacteria	243233
nr	Proteobacteria	Bacteria	323261
pq	Proteobacteria	Bacteria	384676

Table S3 (contd.)

Genome ID	*Phylum*	*Superkingdom*	*NCBI Taxonomy ID*
ps	Proteobacteria	Bacteria	160488
4T	Proteobacteria	Bacteria	205922
pa	Proteobacteria	Bacteria	381754
ri	Proteobacteria	Bacteria	259536
pz	Proteobacteria	Bacteria	335284
tc	Proteobacteria	Bacteria	317025
83	Spirochaetes	Bacteria	456481
9C	Synergistetes	Bacteria	525903
2E	Thermotogae	Bacteria	521045
jt	Thermotogae	Bacteria	484019
63	Crenarchaeota	Archaea	453591
fd	Crenarchaeota	Archaea	490899
2	Crenarchaeota	Archaea	399549
sv	Crenarchaeota	Archaea	273063
0G	Crenarchaeota	Archaea	427318
za	Crenarchaeota	Archaea	330779
34	Crenarchaeota	Archaea	384616
11	Euryarchaeota	Archaea	349307
m9	Euryarchaeota	Archaea	259564
ma	Euryarchaeota	Archaea	188937
mz	Euryarchaeota	Archaea	192952
fu	Euryarchaeota	Archaea	269797
jx	Euryarchaeota	Archaea	521011
m6	Euryarchaeota	Archaea	323259
23	Euryarchaeota	Archaea	368407
mn	Euryarchaeota	Archaea	190192
aQ	Euryarchaeota	Archaea	589924
7W	Euryarchaeota	Archaea	572546
h6	Euryarchaeota	Archaea	523850
tk	Euryarchaeota	Archaea	69014
5V	Euryarchaeota	Archaea	593117
0X	Euryarchaeota	Archaea	604354
ph	Euryarchaeota	Archaea	70601
pb	Euryarchaeota	Archaea	272844
pu	Euryarchaeota	Archaea	186497
ta	Euryarchaeota	Archaea	273075
p3	Euryarchaeota	Archaea	263820
4X	Euryarchaeota	Archaea	485914
3R	Euryarchaeota	Archaea	519442
np	Euryarchaeota	Archaea	348780
4B	Euryarchaeota	Archaea	416348

Table S3 (contd.)

Genome ID	*Phylum*	*Superkingdom*	*NCBI Taxonomy ID*
aG	Euryarchaeota	Archaea	309800
hm	Euryarchaeota	Archaea	272569
3P	Euryarchaeota	Archaea	573064
mj	Euryarchaeota	Archaea	243232
m2	Euryarchaeota	Archaea	444158
57	Euryarchaeota	Archaea	406327
md	Euryarchaeota	Archaea	187420
m4	Euryarchaeota	Archaea	339860
9Q	Euryarchaeota	Archaea	634498
9O	Euryarchaeota	Archaea	439481
